# USGS44, a new high‐purity calcium carbonate reference material for *δ* ^13^C measurements

**DOI:** 10.1002/rcm.9006

**Published:** 2021-01-07

**Authors:** Haiping Qi, Heiko Moossen, Harro A.J. Meijer, Tyler B. Coplen, Anita T. Aerts‐Bijma, Lauren Reid, Heike Geilmann, Jürgen Richter, Michael Rothe, Willi A. Brand, Blaza Toman, Jacqueline Benefield, Jean‐François Hélie

**Affiliations:** ^1^ U.S. Geological Survey Reston VA USA; ^2^ Max Planck Institute for Biogeochemistry Jena Germany; ^3^ Centre for Isotope Research (CIO) University of Groningen Groningen The Netherlands; ^4^ National Institute of Standards and Technology (NIST) Gaithersburg MD USA; ^5^ Centre de recherche Geotop, Département des sciences de la Terre et de l'atmosphère Université du Québec à Montréal Canada

## Abstract

**Rationale:**

The stable carbon isotopic (*δ* ^13^C) reference material (RM) LSVEC Li_2_CO_3_ has been found to be unsuitable for *δ* ^13^C standardization work because its *δ* ^13^C value increases with exposure to atmospheric CO_2_. A new CaCO_3_ RM, USGS44, has been prepared to alleviate this situation.

**Methods:**

USGS44 was prepared from 8 kg of Merck high‐purity CaCO_3_. Two sets of *δ* ^13^C values of USGS44 were determined. The first set of values was determined by online combustion, continuous‐flow (CF) isotope‐ratio mass spectrometry (IRMS) of NBS 19 CaCO_3_ (*δ* ^13^C_VPDB_ = +1.95 milliurey (mUr) exactly, where mUr = 0.001 = 1‰), and LSVEC Li_2_CO_3_ (*δ* ^13^C_VPDB_ = −46.6 mUr exactly), and normalized to the two‐anchor *δ* ^13^C_VPDB‐LSVEC_ isotope‐delta scale. The second set of values was obtained by dual‐inlet (DI)‐IRMS of CO_2_ evolved by reaction of H_3_PO_4_ with carbonates, corrected for cross contamination, and normalized to the single‐anchor *δ* ^13^C_VPDB_ scale.

**Results:**

USGS44 is stable and isotopically homogeneous to within 0.02 mUr in 100‐μg amounts. It has a *δ* ^13^C_VPDB‐LSVEC_ value of −42.21 ± 0.05 mUr. Single‐anchor *δ* ^13^C_VPDB_ values of −42.08 ± 0.01 and −41.99 ± 0.02 mUr were determined by DI‐IRMS with corrections for cross contamination.

**Conclusions:**

The new high‐purity, well‐homogenized calcium carbonate isotopic reference material USGS44 is stable and has a *δ* ^13^C_VPDB‐LSVEC_ value of −42.21 ± 0.05 mUr for both EA/IRMS and DI‐IRMS measurements. As a carbonate relatively depleted in ^13^C, it is intended for daily use as a secondary isotopic reference material to normalize stable carbon isotope delta measurements to the *δ* ^13^C_VPDB‐LSVEC_ scale. It is useful in quantifying drift with time, determining mass‐dependent isotopic fractionation (linearity correction), and adjusting isotope‐ratio‐scale contraction. Due to its fine grain size (smaller than 63 μm), it is not suitable as a *δ* ^18^O reference material. A *δ* ^13^C_VPDB‐LSVEC_ value of −29.99 ± 0.05 mUr was determined for NBS 22 oil.

## 1 INTRODUCTION

High accuracy measurements of stable carbon isotope ratios (*δ* ^13^C values) in naturally occurring materials are necessary in an increasing number of fields, including oceanography, atmospheric sciences, biology, paleoclimatology, geology, environmental sciences, food and drug authentication, and forensic applications. To achieve high‐quality *δ* ^13^C analysis, isotopic reference materials (RMs) are required. In the past several decades, the international isotopic RMs NBS 18, NBS 19, NBS 22, LSVEC, IAEA‐CO‐1, IAEA‐CO‐8, IAEA‐CO‐9, and IAEA‐603 have been gradually introduced to the isotope community and used for the determination of *δ* ^13^C values of carbon‐bearing materials.^1^, ^2^, ^3^ In 1985, the primary recommendation of a Consultants' Group Meeting of the International Atomic Energy Agency (IAEA)^4^ was that a new Vienna Peedee Belemnite (VPDB) *δ* ^13^C scale be established with NBS 19 carbonate assigned the value of +1.95 milliurey (mUr) exactly as its single anchor, where 1 mUr = 0.001 = 1‰.^1^, ^5^ Implementation of this recommendation improved consistency among *δ* ^13^C measurements.^6^ Recognizing that two‐point normalization of the *δ* ^2^H and *δ* ^18^O scales substantially improved agreement among laboratories,^7^ the IAEA convened a panel in 2004 to review stable carbon isotopic RMs and to recommend a second RM for two‐point normalization of the *δ* ^13^C scale. Based on high‐precision isotope‐ratio mass spectrometry (IRMS),^8^, ^9^ a consensus value of −46.6 mUr exactly was assigned to LSVEC lithium carbonate.^10^, ^11^ The results (Table 1 of Coplen et al^10^) were provided to the International Union of Pure and Applied Chemistry (IUPAC). Following recommendations of the Commission on Isotopic Abundances and Atomic Weights (CIAAW) in August 2005 at the 43rd General Assembly of IUPAC in Beijing and recommendations of an IAEA panel, a recommendation evolved that *δ* ^13^C values of all carbon‐bearing materials should be measured and expressed relative to VPDB on a scale normalized by assigning consensus values of −46.6 mUr to LSVEC lithium carbonate and +1.95 mUr to NBS 19 calcium carbonate.^10^, ^11^ The adoption of two‐point normalization improved the standard uncertainties of *δ* ^13^C RMs significantly compared with previously assessed uncertainties, as demonstrated in Figure 1 of Coplen et al.^10^ Since then, determinations of *δ* ^13^C values of most new secondary RMs for forensic, environmental, paleontological, and atmospheric applications^12^, ^13^, ^14^, ^15^, ^16^, ^17^, ^18^, ^19^, ^20^ have been based on the NBS 19‐LSVEC scale with NBS 19 and LSVEC as anchors. An IUPAC technical report, which assessed international RMs for isotope‐ratio measurements, published in 2014 by Brand et al,^1^ tabulates a comprehensive list of *δ* ^13^C values of RMs on the NBS 19‐LSVEC scale.

In 2015, careful laboratory analyses performed at the IAEA, Seibersdorf, Austria, demonstrated that the *δ* ^13^C signature of individual units of LSVEC gradually increased over time (that is, values became less negative) due to contamination with atmospheric CO_2_, and a similar observation was made of the LSVEC material stored at the National Institute of Standards and Technology (NIST, Gaithersburg, MD, USA).^21^, ^22^ Subsequently, this observation was confirmed by Qi et al.^14^ Thus, LSVEC no longer meets minimum requirements for use as a *δ* ^13^C RM, particularly as a scale anchor, and IUPAC has advised against its use as a *δ* ^13^C RM.^23^ However, LSVEC remains satisfactory for use as a lithium isotopic RM. The *δ* ^13^C instability of LSVEC demonstrates the need to develop and characterize a new secondary isotopic RM with the potential to replace LSVEC as the second anchor of the VPDB scale.

The Reston Stable Isotope Laboratory (RSIL, Reston, VA, USA) at the U.S. Geological Survey (USGS) and the stable isotope laboratory at the Max Planck Institute for Biogeochemistry, Jena, Germany (BGC‐IsoLab) surveyed many commercial calcium carbonate reagents and identified one material from Merck that could serve as a secondary *δ* ^13^C RM. This calcium carbonate RM is named USGS44. Due to its fine grain size (<63 μm), it is not suitable as a *δ* ^18^O RM because its oxygen can exchange with atmospheric water, changing its *δ* ^18^O value. Nevertheless, *δ* ^18^O data are reported herein because (1) users may be interested in a nominal *δ* ^18^O value, (2) these data demonstrate the high precision that can be achieved by dual‐inlet IRMS, and (3) these data support the isotopic homogeneity of USGS44. The assessments of *δ* ^13^C values of the material were performed by the RSIL, the BGC‐IsoLab, the Centre for Isotope Research University of Groningen, Groningen, Netherlands (CIO), and the Centre de recherche, Geotop, Université du Québec à Montréal, Canada.

## 2 METHODS

### 2.1 Preparation of USGS44

**FIGURE 1:**
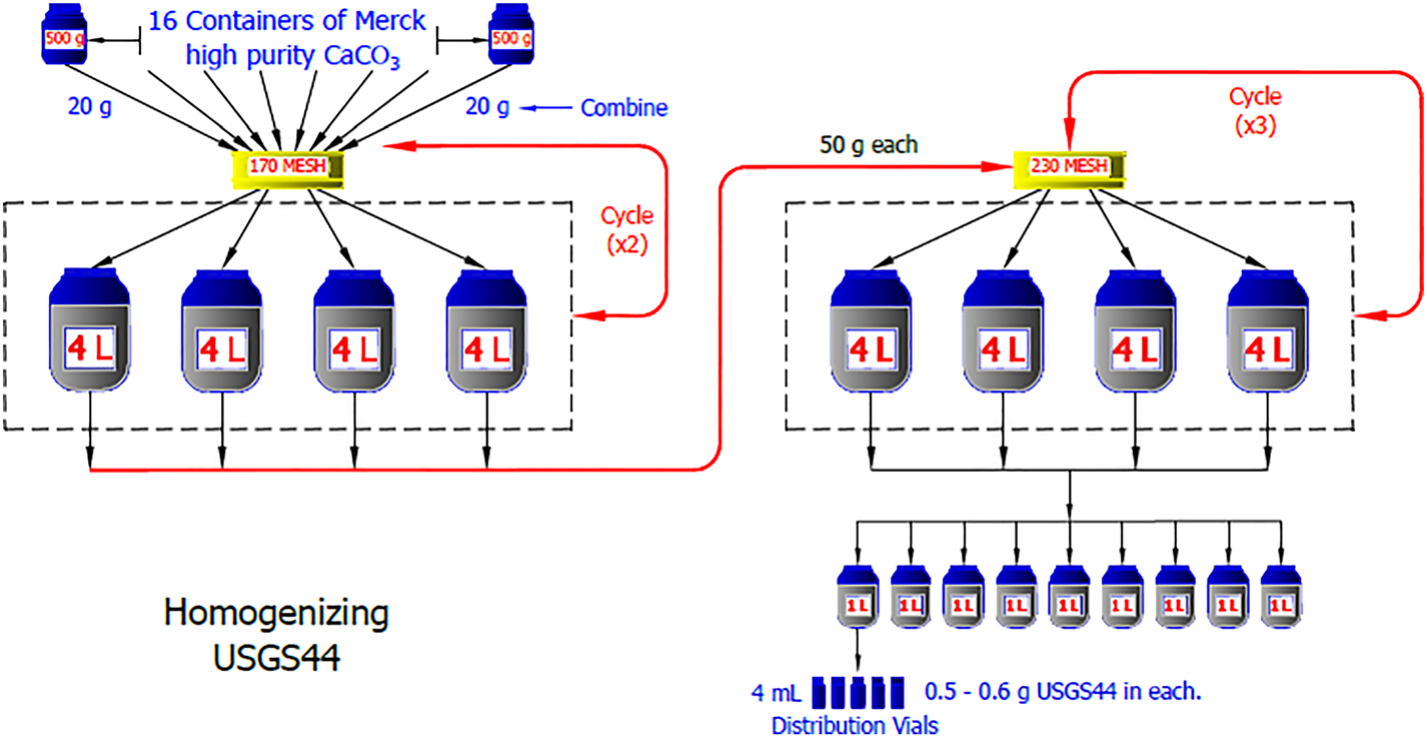
Homogeneity procedure for USGS44

Sixteen bottles of high‐purity calcium carbonate powder with a total mass of 8 kg were purchased from Merck (Darmstadt, Germany). To ensure isotopic homogeneity of the RM, the following steps were carried out (as shown in Figure 1). First, approximately 20 g of material was removed from each of these sixteen 500‐g bottles, combined, and passed through a 170‐mesh (88 μm) stainless steel sieve with an AS200 sieve shaker (Retsch, Newtown, PA, USA) to homogenize the material. The very small amount of material larger than 88 μm was discarded. The sieved material was divided and collected in four 4‐L glass containers. The same steps were repeated until all materials from the original 16 bottles were combined and either passed through the 88‐μm sieve or were discarded after not passing through the sieve. Second, approximately 50 g of material was removed from each of the four 4‐L containers, combined, and passed through a 170‐mesh sieve, mixed, sieved again and distributed evenly in four new 4‐L glass containers. Third, about 50 g of material was taken from each of these four containers, combined, passed through a 230‐mesh (63 μm) stainless steel sieve, distributed among nine new 2‐L glass containers, and repeated until all material passed through the sieve. The third step was repeated three times to thoroughly homogenize the material. Then, samples were taken from the top, middle, and bottom of each of these jars for use in homogeneity testing. The large batch of material was stored in several 1‐L vacuum‐sealed glass flasks. From these flasks, individual aliquots of 0.5–0.6 g each were distributed into 4‐mL glass vials, with Polyseal caps, and vacuum sealed in plastic pouches. All RMs were stored in a cool, dry, dark environment.

### 2.2 Isotopic reference materials used in participating laboratories

The analytical methods used to measure *δ* ^13^C values are unique for each laboratory according to instrumentation and experience. The internationally distributed RMs used in this study included NBS 19 CaCO_3_, IAEA‐603 CaCO_3_, NBS 22 oil, and LSVEC Li_2_CO_3_. These are among the RMs listed in Table 1 along with their most up‐to‐date *δ* ^13^C values, associated uncertainties, and sources for the values provided. Although LSVEC exhibits issues discussed in the introduction above,^14^, ^21^, ^22^ a second scale anchor that is independent of LSVEC currently does not exist. In this study, we still analyzed and used LSVEC for normalization. To ensure the best quality possible, we used fresh aliquots from the NIST stock material that served to determine the *δ* ^13^C_VPDB_ value of USGS41a.^14^ We also noticed that *δ* ^13^C values of LSVEC can be significantly more negative when analyzed using the classical acid digestion method than the values obtained using the elemental analyzer (EA) technique. The observation has not found a satisfactory explanation so far and warrants further experimental investigations. NBS 22 oil, which was anchored to LSVEC, is used as an anchor in this study.^10^, ^11^ The use of NBS 22 oil, which was crimp‐sealed in silver tubes,^28^ made it possible to measure *δ* ^13^C values of USGS44 directly on an EA connected to an isotope‐ratio mass spectrometer following the principle of identical treatment.^29^ We are aware that IAEA‐603 exhibits inhomogeneities at microgram analysis^30^; however, this problem does not affect the current study due to the relatively large sample amounts (about 0.2–40 mg) used.

**TABLE 1:** *δ* ^13^C values of international standards scaled to the VPDB‐LSVEC scale and scaled to a USGS44 *δ* ^13^C_VPDB_ value of −42.08 mUr and −41.99 mUr. [Values used for two‐point *δ* ^13^C normalization of scales are shown in bold. BCG‐IsoLab and CIO normalized values are shown in columns 4 and 5, respectively. The uncertainties listed in column 6 are those provided in the cited literature. These uncertainties are often – especially for certified materials – Expanded uncertainties (*U*) of combined standard uncertainties (*u*_c_) with a coverage factor *k* = 2 (*U* = *k*·*u*_c_). Data in the scientific literature provide a larger variety of uncertainties and, in many cases, the measurement precision alone, usually expressed as 1‐sigma value. The type of uncertainty is not stated in the tables. For further information, readers should consult the original literature. *, determined in this study]

Reference material ID	Substance	*δ* ^13^C_VPDB‐LSVEC_ (mUr)	*δ* ^13^C_VPDB_ scaled to −42.08 mUr for USGS44 (mUr)	*δ* ^13^C_VPDB_ scaled to −41.99 mUr for USGS44 (mUr)	Literature values (pre‐VPDB‐LSVEC era) (mUr)	Citation
IAEA‐CO‐1	Calcite	**+**2.48	+2.48	+2.48	+2.48 ± 0.03	Stichler^6^
IAEA‐603	Calcite	+2.46	+2.46	+2.46	None	Assonov et al^3^
NBS 19	Limestone	**+1.95**	**+1.95**	**+1.95**	+1.95 exactly	Friedman et al,^24^ Hut^4^
RM 8562	Carbon dioxide	−3.72	−3.70	−3.69	−3.72 ± 0.04	Verkouteren and Klinedinst^9^
NBS 18	Carbonatite	−5.01	−4.99	−4.98	−5.01 ± 0.03	Friedman et al^24^
IAEA‐CO‐8	Calcite	−5.76	−5.74	−5.72	−5.75 ± 0.06	Stichler^6^
IAEA‐CH‐6	Sucrose	−10.45	−10.41	−10.39	−10.43 ± 0.13	Gonfiantini et al^25^
RM 8564	Carbon dioxide	−10.45	−10.41	−10.39	−10.45 ± 0.03	Verkouteren and Klinedinst^9^
USGS24	Graphite	−16.05	−16.00	−15.96	−15.99 ± 0.11	Gonfiantini et al^25^
IAEA‐CH‐3	Cellulose	−24.72	−24.64	−24.59	None	
USGS40	l‐glutamic acid	−26.39	−26.23	−26.17	−26.24	Qi et al^26^
IAEA‐600	Caffeine	−27.77	−27.68	−27.62	None	
IAEA‐601	Benzoic acid	−28.81	−28.72	−28.66	None	
IAEA‐602	Benzoic acid	−28.85	−28.76	−28.70	None	
NBS 22	Oil	−29.99*	−29.90	−29.83	−29.91 ± 0.03 −29.95 ± 0.05	Qi et al,^26^ Stalker et al^27^
IAEA‐CH‐7	Polyethylene foil	−32.15	−32.05	−31.98	−31.83 ± 0.11	Gonfiantini et al^25^
RM 8563	Carbon dioxide	−41.59	−41.46	−41.37	−41.57 ± 0.09	Verkouteren and Klinedinst^9^
USGS44	Calcium carbonate	−42.21*	−42.08*	−41.99*	None	
LSVEC	Lithium carbonate	**−46.6**	−46.46	−46.36	−46.48 ± 0.15	Stichler^6^
IAEA‐CO‐9	Barium carbonate	−47.32	−47.17	−47.07	−47.12 ± 0.15	Stichler^6^

### 2.3 Online combustion continuous‐flow IRMS

At the RSIL, the methods used for online *δ* ^13^C analysis are similar to the procedures and techniques used previously for determination of *δ* ^13^C values of secondary *δ* ^13^C RMs.^14^ The USGS44 RM and internationally distributed RMs were analyzed on two different elemental analyzers (EAs) (ECS 4010; Costech, Valencia, CA, USA, and EA Isolink; Thermo Fisher Scientific, Bremen, Germany). Both EAs were connected to a ConFlo IV interface (Thermo Fisher Scientific), which was connected to a Delta V isotope‐ratio mass spectrometer (Thermo Fisher Scientific). The *δ* ^13^C_VPDB_ values of USGS44 were normalized to NBS 19 calcium carbonate (*δ* ^13^C = +1.95 mUr exactly) and LSVEC (*δ* ^13^C = −46.6 mUr exactly).

At BGC‐IsoLab, the measurement procedures for *δ* ^13^C EA‐IRMS analyses largely followed the described procedures and techniques from previous publications.^29^, ^31^, ^32^, ^33^ USGS44 samples from six different aliquots were analyzed using a Delta^plus^ isotope‐ratio mass spectrometer (Thermo Fisher Scientific) coupled to a 1100 CE EA analyzer (Carlo Erba, Rodano, Italy) via a ConFlo III open‐split interface (Thermo Fisher Sceintific). In most cases, the samples were analyzed in dilution mode to reduce systematic errors associated with blanks. Measurement sequences and post measurement blank, linearity, and drift corrections were performed according to Werner and Brand.^29^ The *δ* ^13^C_VPDB_ values of USGS44 were normalized by assignment of IAEA‐603 calcium carbonate *δ* ^13^C = +2.46 mUr^2^ (or NBS 19 *δ* ^13^C = +1.95 mUr) and LSVEC *δ* ^13^C = −46.60 mUr.

At Geotop, 2.8 ± 0.1 mg of CaCO_3_ were weighed into tin cups to obtain the same amount of CO_2_ for all samples and RMs, hence eliminating potential linearity issues. The samples were then analyzed with an Isoprime 100 isotope‐ratio mass spectrometer (Micromass, now Elementar UK Ltd, Cheadle, UK) coupled to a Vario MicroCube elemental analyzer (Elementar, Langenselbold, Germany) in continuous‐flow mode. The samples were analyzed in dilution mode. Blank corrections were performed according to Werner and Brand.^29^ No drift was observed. The *δ* ^13^C_VPDB_ values of USGS44 were normalized by assignment of NBS 19 calcium carbonate *δ* ^13^C = +1.95 mUr and LSVEC *δ* ^13^C = −46.6 mUr exactly. All reference materials were stored under vacuum.

### 2.4 Offline dual‐inlet IRMS

Cross contamination between the fraction of reference gas that contaminates the sample, and vice versa, must be accounted for during dual‐inlet measurements. The symbol of the dimensionless quantity used to express cross contamination is *η* (eta), and it is a property of a specific dual‐inlet isotope‐ratio mass spectrometer during a specified time.^34^ It is dependent upon the instrumental settings under which measurements are performed.^8^, ^34^ For some analytical runs IAEA‐603 was used as an anchor with an assigned *δ* ^13^C_VPDB_ = +2.46 mUr and *δ* ^18^O_VPDB_ of solid IAEA‐603 = −2.37 mUr.^2^

**FIGURE 2:**
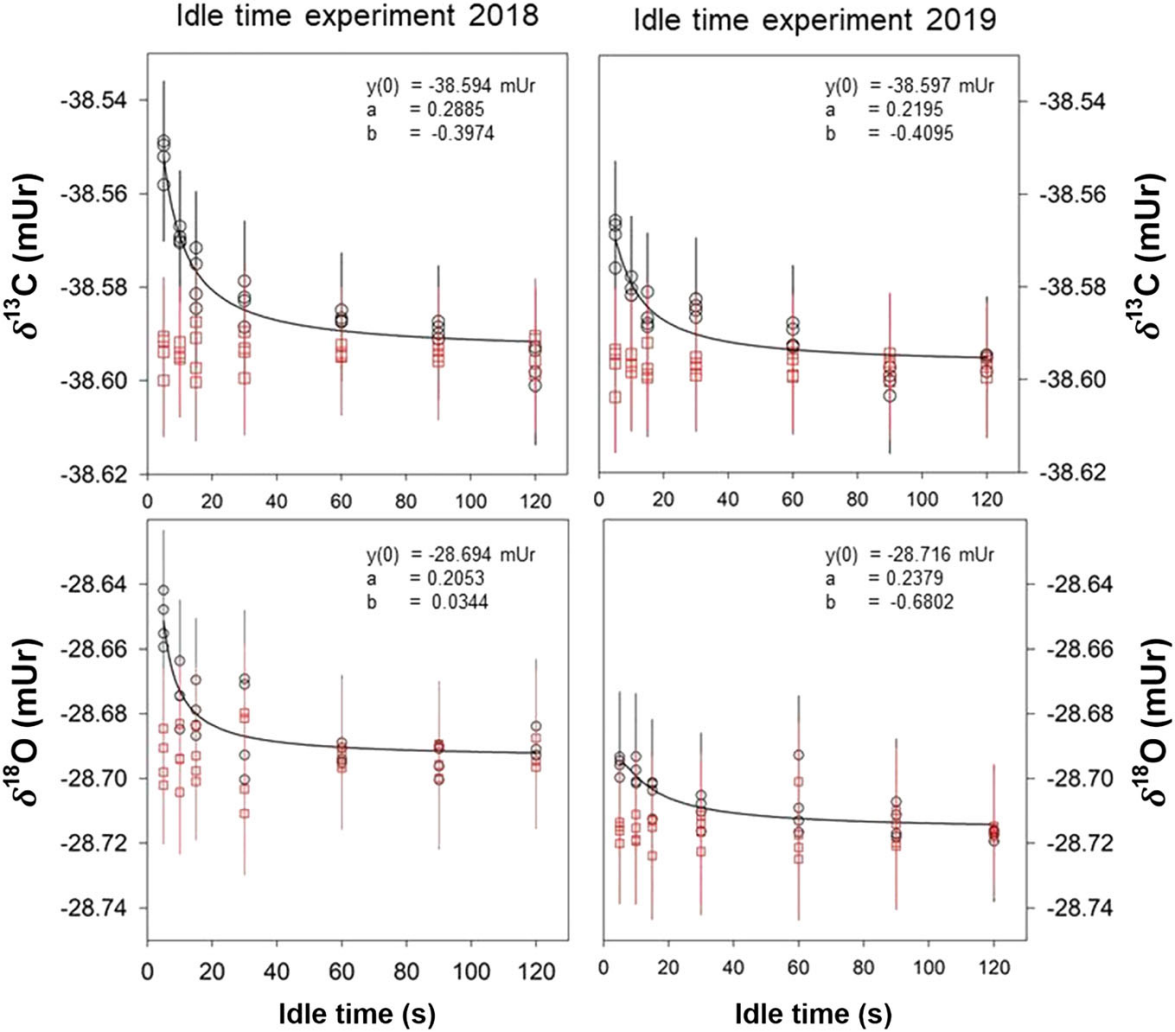
Idle time experiments conducted 1 year apart on the MAT 253 dual‐inlet isotope‐ratio mass spectrometer at BGC‐IsoLab. Black circles and expanded uncertainty bars indicate results which have not been corrected for cross contamination. An inverse second‐order polynomial equation, ($f=y\left(0\right)+\frac{a}{x}+\frac{b}{x^{2}}$), was fitted to all datasets with the value of *y*(0) being taken as the true delta value “*δ*_T_”. The red squares with error bars indicate cross‐contamination corrected results relative to the idle time. The values of *η* for an idle time of 15 s are 0.00046 ± 0.0005 and 0.00097 ± 0.001 in 2018, and 0.00032 ± 0.0005 and 0.00078 ± 0.001 in 2019, for the *δ* ^13^C and *δ* ^18^O values of CO_2_, respectively

BGC‐IsoLab evolved CO_2_ from the USGS44, IAEA‐603, and NBS 19 calcium carbonates using its “*A*cid *R*eaction *a*nd *Mi*xing *S*ystem” (ARAMIS). We refer the reader to the relevant publications for details on ARAMIS and the reaction procedure.^29^, ^33^, ^35^ CO_2_ was evolved from four aliquots of USGS44 (and other RMs) at a reaction temperature of 25 ± 0.1°C, frozen into 300‐mL sample vials, and analyzed on a MAT 253 isotope‐ratio mass spectrometer (Thermo Fisher Scientific) equipped with a dual‐inlet system. Because no second‐scale anchor is available for such measurements, it is paramount to avoid scale contraction effects. Thus, ion source settings were chosen to minimize the value of *η*, and idle time experiments were conducted in 2018 and 2019 to evaluate *η* and its stability over time (Figure 2). A subset of USGS44 CO_2_ gas samples was analyzed on an older MAT 252 DI‐IRMS instrument that is known to have minimal cross contamination^8^ to verify the value of *η* for the MAT 253 isotope‐ratio mass spectrometer. IUPAC‐recommended ^17^O correction parameters (*λ*: 0.528; *K*: 0.01027689)^36^ and the SSH algorithm were used in the Isodat software (Thermo Fisher Scientific) for the online ^17^O correction. Offline data evaluation included daily drift correction and normalization to the *δ* ^13^C_VPDB_ scale on the MAT 252 IRMS measurements. In addition to those corrections, an offline *η* correction was necessary for the MAT 253 measurements.

At CIO, CO_2_ evolved by treatment of USGS44, NBS 19, and IAEA‐603 calcium carbonates with high‐purity phosphoric acid at 25.0 ± 0.1°C was analyzed with a dual‐inlet MM10 isotope‐ratio mass spectrometer (Micromass Ltd, now Elementar UK Ltd). Measurements and determination of the value of *η* were performed as described in Meijer et al^34^ and Meijer.^37^ Ion source settings were selected to minimize the value of *η*. Offline data evaluation included daily drift correction and normalization to the *δ* ^13^C_VPDB_ scale. IUPAC‐recommended ^17^O correction parameters (*λ*: 0.528; *K*: 0.01027689)^36^ and the SSH algorithm were used in the data reduction for the online ^17^O correction. A varying number of samples of USGS44 were extracted during the same time period as at BGC‐IsoLab (three in 2016, four in 2017, eight in 2018, and ten in 2019). Each separate acid reaction corresponds to one sample. In addition, the 2017 and 2018 sample sets contained LSVEC, and the 2018 and 2019 sets contained IAEA‐603. All samples were calibrated using CO_2_ evolved from NBS 19 or IAEA‐603, of which the same number of aliquots were produced and measured.

### 2.5 GasBench and MultiCarb

At the RSIL, a GasBench II gas preparation and introduction system (ThermoFinnigan, now Thermo Fisher Scientific) – equipped with a PAL autosampler (CTC Analytics AG, Zwingen, Switzerland) coupled to a ConFlo IV interface and a Delta XP isotope‐ratio mass spectrometer (both Thermo Fisher Scientific) – was used to check the isotopic homogeneity of USGS44 at microgram masses. The method used was modified from Breitenbach and Bernasconi.^38^ About 100–200 μg of CaCO_3_ were weighed and loaded into 12‐mL round‐bottomed borosilicate vials (Exetainers, Labco, High Wycombe, UK, Part No. 938 W) capped with Labco butyl rubber septa. The vials were flushed for 10 min on an in‐house multiple‐port purging line with grade 5.0 helium at a flow rate of 55 mL/min so that no air contamination was observed during our experiments. Ten drops of 102% H_3_PO_4_ were injected into each vial to react with the calcium carbonate. The Exetainers were placed in the aluminum block of the GasBench II and heated to 25 ± 0.1°C overnight to ensure quantitative conversion into CO_2_.

At Geotop, between 100 and 120 μg of NBS 19, IAEA‐603, LSVEC, and USGS44 were weighed into glass micro crucibles. The samples were then transferred into glass conical‐bottomed vials closed with septum caps. The samples were inserted in a 90°C heated rack. After a minimum of 1 h of heating, samples were analyzed with an Isoprime isotope‐ratio mass spectrometer coupled to a Isoprime MultiCarb preparation system in dual‐inlet mode. For each sample, three drops of ortho‐phosphoric acid (*ρ* = 1.92 g/cm^3^) were delivered under vacuum. The resulting CO_2_ was trapped in a cold finger at −180°C (liquid nitrogen) for 15 min. A water trap (−70°C) was used to condense any moisture between the vial and the cold finger. The “dry” CO_2_ was then heated at −60°C and “focused” in a second cold finger at −160°C for 5 min. The resulting gas was released in a fixed volume and the pressure of the “monitoring gas” was equilibrated with that of the sample. The “monitoring gas” is a Jackson Dome CO_2_ with an approximate *δ* ^13^C value of −3 mUr. Because the original design of the measurement sequence included LSVEC, no cross‐contamination test was performed. IUPAC‐recommended ^17^O correction parameters (*λ*: 0.528; *K*: 0.01027689)^36^ and the SSH algorithm were used in the IonVantage software (Elementar) for the online ^17^O correction.

### 2.6 Weighing NBS 22 oil

At RSIL, GF/C glass microfiber filters (Whatman, Maidstone, UK) were used to weigh NBS 22 oil. Prior to weighing, the glass filters were baked at 475°C for 2 h. The baked filters were cut into 1.5 × 1.5‐mm pieces. A small piece of filter was placed on an unfolded 5 × 3.5‐mm tin capsule. A clean, thin stainless‐steel wire was used to transfer a tiny drop of oil onto the glass filter. The weighed oil on the filter was wrapped into a tin capsule.

## 3 RESULTS AND DISCUSSION

### 3.1 Homogeneity evaluation

At the RSIL, the homogenized USGS44 was divided and stored in nine glass containers. Three aliquots of USGS44 were sampled from the top, middle, and bottom of each container, making a total of 27 samples for isotopic homogeneity evaluation. The homogeneity test was carried out by measuring *δ* ^13^C using two different EAs and a GasBench at the RSIL and a MultiCarb system at Geotop with different masses of USGS44 as specified above. At the RSIL, an IsoLink EA was used, and three aliquots containing 84 μg of carbon (0.70 mg of calcium carbonate) from each of 27 fractions were analyzed to confirm *δ* ^13^C homogeneity. Homogeneity tests were carried out in four separate analytical sequences. The first fraction of USGS44 was designated as a quality control (QC) sample and was analyzed at the beginning, middle, and end of each of the four sequences. The measured *δ* ^13^C values along with associated 1‐σ standard deviations of USGS44 are summarized in Table 2. The *δ* ^13^C values in this table were normalized to the QC sample by assigning it a *δ* ^13^C value of −42.21 mUr. The uncertainty from nine bottles of USGS44 was 0.03 mUr, which indicates that the USGS44 is well homogenized at amounts of 0.70 mg. The overall standard deviation from 78 individual analyses is 0.05 mUr. In a second RSIL test, a Costech EA, as specified above, was used. One aliquot containing 12 μg carbon (0.10 mg of calcium carbonate) from each of 27 fractions was analyzed. In this sequence, NBS 19 was analyzed at the beginning, middle, and end as a control sample, and IAEA‐603 was also analyzed. The measured *δ* ^13^C values of the 0.10‐mg samples of USGS44 are presented in Table 3 and they demonstrate that the data quality is comparable with that of NBS 19 and IAEA‐603. The overall standard deviation of 0.07 mUr from the nine bottles with 26 analyses at 0.10‐mg of USGS44 is slightly higher than 0.05 mUr from 0.70‐mg analyses of USGS44, and this is thought to be caused by a variable carbon blank from the tin capsules. The average *δ* ^13^C value of −40.08 mUr from USGS44 in Table 3 is the result of single‐point normalization against NBS 19.

**TABLE 2:** Measured *δ* ^13^C values from the homogeneity tests of USGS44 by EA/IRMS with sample masses of 0.70 mg CaCO_3_. [Normalized to QC samples by assigning *δ* ^13^C of USGS44 to −42.21 mUr. Uncertainties listed are 1‐σ standard deviations]

USGS44 vials	Bottle A *δ* ^13^C (mUr)	Bottle B *δ* ^13^C (mUr)	Bottle C *δ* ^13^C (mUr)	Bottle D *δ* ^13^C (mUr)	Bottle E *δ* ^13^C (mUr)	Bottle F *δ* ^13^C (mUr)	Bottle G *δ* ^13^C (mUr)	Bottle H *δ* ^13^C (mUr)	Bottle I *δ* ^13^C (mUr)
Top	−42.24	−42.18	−42.28	−42.20	−42.24	−42.23	−42.23	−42.21 ±	−42.21
	± 0.07	± 0.01	± 0.04	± 0.03	± 0.07	± 0.03	± 0.04	0.08	± 0.03
	n = 3	n = 3	n = 3	n = 3	n = 3	n = 3	n = 3	n = 3	n = 3
Middle	−42.23	−42.23	−42.22	−42.20	−42.21	−42.19	−42.15	−42.20	−42.23
	± 0.02	± 0.03	± 0.08	± 0.06	± 0.04	± 0.03	± 0.07	± 0.04	± 0.03
	n = 3	n = 3	n = 2	n = 3	n = 3	n = 3	n = 3	n = 3	n = 3
Bottom	−42.23	−42.18	−42.28	−42.20	−42.20	−42.20	−42.24	−42.23	−42.20
	± 0.10	± 0.04		± 0.04	± 0.07	± 0.06	± 0.02	± 0.02	± 0.08
	n = 3	n = 3	n = 1	n = 3	n = 3	n = 3	n = 3	n = 3	n = 3
Average	−42.23	−42.19	−42.26	−42.20	−42.22	−42.21	−42.21	−42.21	−42.21
	± 0.06	± 0.03	± 0.06	± 0.04	± 0.06	± 0.04	± 0.06	± 0.05	± 0.05
Grand average	−42.21 ± 0.03 mUr, n = 27								

**TABLE 3:** Measured *δ* ^13^C values from the homogeneity tests of USGS44 with NBS 19 and IAEA‐603 by EA/IRMS. [Sample masses are 0.10 mg. Normalized NBS 19 with *δ* ^13^C = +1.95 mUr. Uncertainties listed are 1‐σ standard deviations]

Bottle A *δ* ^13^C (mUr)	Bottle B *δ* ^13^C (mUr)	Bottle C *δ* ^13^C (mUr)	Bottle D *δ* ^13^C (mUr)	Bottle E *δ* ^13^C (mUr)	Bottle F *δ* ^13^C (mUr)	Bottle G *δ* ^13^C (mUr)	Bottle H *δ* ^13^C (mUr)	Bottle I *δ* ^13^C (mUr)	NBS 19 *δ* ^13^C (mUr)	IAEA‐603 *δ* ^13^C (mUr)
−40.03	−40.13	−40.18	−40.06	−39.98	−40.06	−39.99	−40.15	−40.14	+1.95	+2.47
± 0.05	± 0.07	± 0.08	± 0.18	± 0.04	± 0.09	± 0.01	± 0.13	± 0.05	± 0.10	± 0.06
n = 3	n = 3	n = 3	n = 3	n = 3	n = 3	n = 2	n = 3	n = 3	n = 12	n = 6
Average *δ* ^13^C of USGS44 from 9 bottles: −40.08 ± 0.07 (n = 9)										

Considering the need for high‐precision *δ* ^13^C analysis to normalize small samples, such as in the analysis of foraminifera^39^, ^40^ and basalt,^41^ carbonate RMs must be well homogenized.^30^ Several studies have demonstrated that using an isotope‐ratio mass spectrometer with a GasBench can achieve high‐precision *δ* ^13^C analyses,^38^, ^40^, ^41^, ^42^, ^43^, ^44^, ^45^ and methods for analyzing microgram masses of carbonate have been in use for decades.^38^, ^41^, ^43^ For this reason, we carried out homogeneity tests with a GasBench as specified above at the RSIL and a MultiCarb system at Geotop. At the RSIL, one sample from the middle of each of the nine bottles (total of 9 vials) of USGS44 was analyzed at 0.1‐mg mass level along with NBS 19 and IAEA‐603. A 0.02‐mUr reproducibility (Table 4) of USGS44 indicates that this material is well homogenized. A 0.05‐mUr reproducibility obtained by the MultiCarb system (Table 4) from a randomly selected USGS44 vial with a sample mass of 0.1 to 0.2 mg also confirms that USGS44 is isotopically homogeneous. The average *δ* ^13^C value of −41.94 mUr from USGS44 in Table 4 also is the result of single‐point normalization with NBS 19.

**TABLE 4:** Measured *δ* ^13^C values from homogeneity tests of USGS44 with NBS 19, IAEA‐603, and IAEA‐CO‐1 with a GasBench and MultiCarb system [n.d., not determined; sample masses range from 0.1 to 0.2 mg. Normalized to single RM NBS 19 with *δ* ^13^C = +1.95 mUr. Uncertainties listed are 1‐σ standard deviations]

Method	Mass (mg)	USGS44 *δ* ^13^C (mUr)	NBS 19 *δ* ^13^C (mUr)	IAEA‐603 *δ* ^13^C (mUr)	IAEA‐CO‐1 *δ* ^13^C (mUr)
GasBench RSIL run 1	0.1	−42.08 ± 0.02 (n = 9)	+1.95 ± 0.04 (n = 7)	+2.48 ± 0.07 (n = 9)	n.d.
GasBench RSIL run 2	0.2	−41.65 ± 0.02 (n = 4)	+1.95 ± 0.07 (n = 4)	+2.47 ± 0.02 (n = 4)	+2.42 ± 0.08 (n = 4)
GasBench RSIL run 3	0.2	−42.14 ± 0.01 (n = 6)	+1.95 ± 0.02 (n = 6)	n.d.	n.d.
MultiCarb Geotop run 1	0.1 to 0.2	−41.90 ± 0.05 (n = 4)	+1.95 ± 0.01 (n = 4)	+2.47 ± 0.03 (n = 4)	n.d.
Average		−41.94 ± 0.22 (n = 4)		+2.47 ± 0.01 (n = 3)	

**FIGURE 3:**
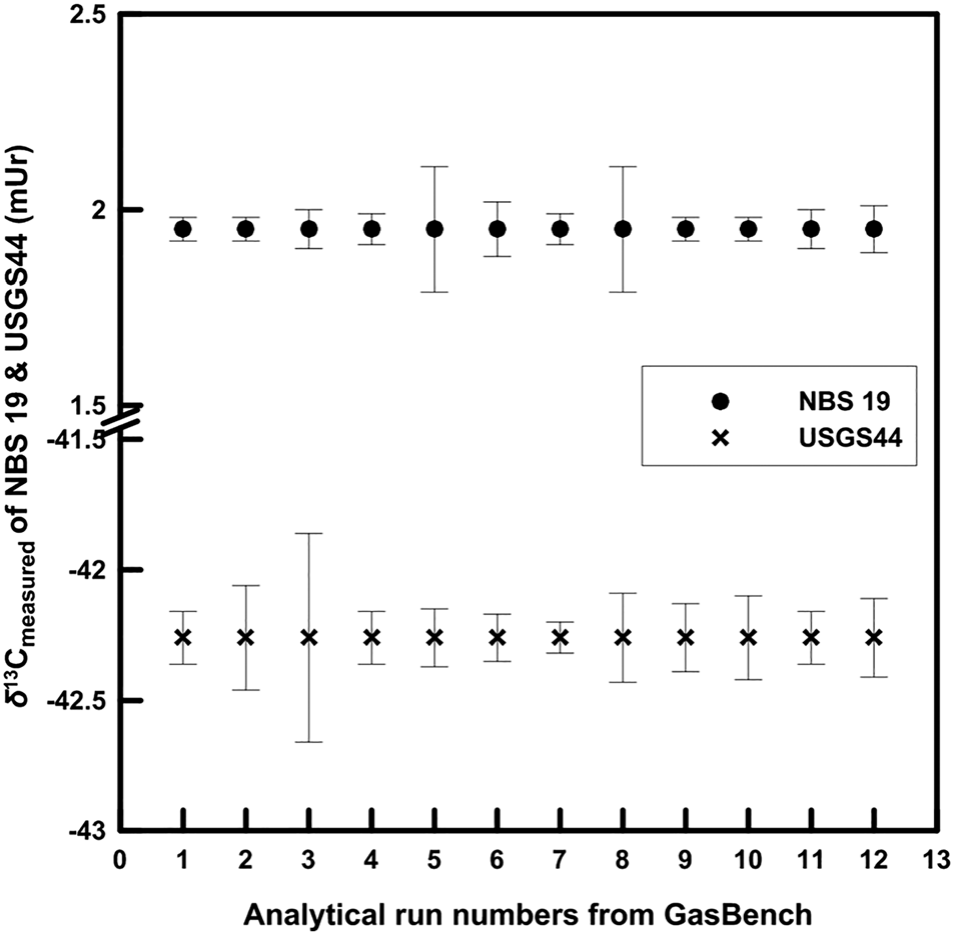
Measured *δ* ^13^C values of NBS 19 and USGS44 on the GasBench from 12 analytical‐run sequences between October 2019 and February 2020. The mass of samples ranges from 0.10 mg to 0.40 mg. Uncertainty bars represent 1‐σ standard deviation of 3 to 10 individual analyses of a single run

The isotopic homogeneity of USGS44 was also evaluated in comparison with NBS 19 using the data from routine sample analysis with the GasBench at the RSIL. Figure 3 shows the data quality from 12 analytical runs between October 2019 and February 2020. The error bars represent 1‐σ standard deviation of an average value of 3 to 10 analyses. The masses ranged between 0.10 mg and 0.40 mg of calcium carbonate. The average standard deviation of 0.15 mUr from USGS44 is higher than that of 0.06 mUr of NBS 19. Evaluating the uncertainties from USGS44 and NBS 19 analyzed by EA/IRMS (Table 3) at the 0.10‐mg level, the higher uncertainty of USGS44 obtained with the GasBench probably does not reflect the true material homogeneity, but rather the uncertainty of the analytical method. We suspect that small variable amounts of atmospheric CO_2_ (*δ* ^13^C_VPDB_ value ~ −8 mUr) were introduced into sample vials when purging the samples, which affects the *δ* ^13^C value of USGS44 (*δ* ^13^C = −42.21 mUr) more than that of NBS 19 (+1.95 mUr).

### 3.2 *δ* ^13^C stability evaluation

To ensure that USGS44 calcium carbonate is a stable material and that its *δ* ^13^C value does not change when the material is exposed to a humid environment,^14^, ^21^, ^22^ a CO_2_ equilibration test^14^ like that carried out with LSVEC was performed with USGS44. Two Merck CaCO_3_ samples (with different lot numbers) were selected for this test. One was a 2‐g vial of CaCO_3_, and another was a large bottle containing 500 g of CaCO_3_, which was the candidate material for USGS44. Three aliquots of about 1‐g of each material were loaded into an 8‐L glass desiccator. For comparison, a set of LSVEC samples was also placed in the CO_2_ equilibration desiccator along with CaCO_3_. A vial of water was also placed inside the desiccator to ensure a humid environment. The desiccator was evacuated and approximately 300 μmol of CO_2_ (*δ* ^13^C = −4 mUr) was introduced into the desiccator. After 7 days at ambient temperature, the samples were removed from the desiccator and dried in a vacuum oven at 40°C for 5 h. Comparison measurements between original samples and samples that had been equilibrated with CO_2_ were made in the same analytical sequence. The measured *δ* ^13^C values are shown in Table 5, and demonstrate that LSVEC reacted with CO_2_ and its *δ* ^13^C value increased by 1.01 mUr. This confirms previous observations that the *δ* ^13^C value of LSVEC is not stable.^14^, ^21^, ^22^ There was no evidence of reaction or exchange between CO_2_ and the two Merck CaCO_3_ materials, which demonstrates that USGS44 is stable and acceptable for use as a *δ* ^13^C RM.

**TABLE 5:** Measured *δ* ^13^C values of CO_2_ exchanged and of non‐exchanged RMs. [Normalized to non‐equilibrated LSVEC by assigning its *δ* ^13^C value as −46.6 mUr. Uncertainties listed are 1‐σ standard deviations]

Treatment	Merck 2‐g vial (lot #: B0759859 251) (mUr)	Merck 500‐g bottle (lot #: B1164559 615) (mUr)	LSVEC (mUr)
Not equilibrated with CO_2_	−49.69 ± 0.01	−42.18 ± 0.02	−46.60 ± 0.06
	n = 3	n = 3	n = 6
Equilibrated with CO_2_ and dried	−49.68 ± 0.01	−42.16 ± 0.04	−45.59 ± 0.06
	n = 3	n = 3	n = 3
Difference between non‐ equilibrated and equilibrated material	0.01	0.02	−1.01

### 3.3 Evaluation of carbon blanks

At the RSIL, the carbon blanks from both the tin capsule and the glass filter were carefully evaluated against USGS40 l‐glutamic acid. Six to eight 5 × 3.5‐mm tin capsules were folded together to act as one sample to produce a substantial CO_2_ peak so that the carbon blank and the *δ* ^13^C value of the blank could be determined accurately. A *δ* ^13^C_VPDB_ value of −26.0 mUr, normalized to USGS40, was obtained for the blank of the tin capsules. The carbon blank in each capsule was about 1 μg. The carbon blank is thought to be a byproduct of mineral oil used in the production of the tin capsules, causing the blanks to be similar within the same batch of capsules. Using the same method, six 1.5 × 1.5‐mm baked glass filters were combined to act as one sample. The CO_2_ peak from the glass filters was too small to integrate as a peak. Therefore, the *δ* ^13^C values were only corrected for the carbon blank introduced from use of tin capsules. A similar carbon blank evaluation was carried out at BGC‐IsoLab. The smaller, but heavier smooth‐wall tin capsules for liquid samples, and the larger, but lighter, tin capsules for standard solid samples were investigated by combining 10 tin capsules into one larger sample and burning that to obtain a satisfactorily sized blank peak. In both cases a value of approximately −27 mUr was obtained for the *δ* ^13^C_VPDB_ of the carbon blank. The amount of blank was so small that the blank‐corrected USGS44 value deviated by less than 0.02 mUr.

### 3.4 Evaluation of quantitative conversion with different sample matrices

To ensure that the determination of the *δ* ^13^C_VPDB_ value of UGSS44 is traceable to the VPDB scale, the primary stable carbon isotopic RMs NBS 19 (*δ* ^13^C_VPDB_ = +1.95 mUr exactly)^4^ and IAEA‐603 (*δ* ^13^C_VPDB_ = +2.46 ± 0.01 mUr)^2^, ^3^ were used. To apply a scale correction to determine the *δ* ^13^C_VPDB_ value of USGS44, NBS 22 oil was used as the second anchor point with an assumed *δ* ^13^C_VPDB_ value of −30.03 mUr. Because oil and calcite are different chemical matrices, the quantitative conversion of carbon from these two materials was thoroughly investigated. The quantitative conversion of LSVEC Li_2_CO_3_ had been evaluated in the study of the determination of the *δ* ^13^C_VPDB_ value of USGS41a^14^ and was not repeated in this work. The masses of NBS 19, NBS 22, and USGS44 ranged from 102 μg (0.85 mg calcite) to 552 μg (4.6 mg calcite). Samples were analyzed in a single analytical sequence. Three aliquots of each sample of each mass were analyzed. BGC‐IsoLab used NBS 22 weighed into tin capsules to normalize USGS44 values, while the RSIL used NBS 22 weighed and sealed in silver tubes. The measured *δ* ^13^C values from NBS 19, NBS 22, and USGS44, and the normalized *δ* ^13^C_VPDB_ values are summarized in Table 6. NBS 22 oil sealed in silver tubes yielded identical *δ* ^13^C_VPDB_ results to those weighed in tin capsules as long as the sample amounts were identical, which also indicates that the carbon blank from tiny pieces of glass filter used for oil weighing is negligible. The sample amount does not affect the final *δ* ^13^C_VPDB_ values of USGS44 if all samples and standards contain the same amount of carbon, thereby minimizing linearity issues.

**TABLE 6:** Variation in the *δ* ^13^C values of calcium carbonate and NBS 22 oil as a function of mass. [All measurements performed by the RSIL. Uncertainties listed are 1‐σ standard deviations]

Mass of carbonate (mg)	Measured *δ* ^13^C of NBS 19 (mUr)	Treatment	Measured *δ* ^13^C of NBS 22 (mUr)	Measured *δ* ^13^C of USGS44 (mUr)	*δ* ^13^C of USGS44 on a scale normalized such that *δ* ^13^C_VPDB_ of NBS 19 and NBS 22 are +1.95 and −30.03 mUr, respectively (mUr)
0.855	+3.22 ± 0.05	Sealed in Ag tube	−28.41 ± 0.02	−40.54 ± 0.05	−42.25 ± 0.05
		Weighed in tin cup	−28.41 ± 0.02		
1.538	+3.34 ± 0.05	Sealed in Ag tube	−28.49 ± 0.02	−40.64 ± 0.03	−42.24 ± 0.03
		Weighed in tin cup	−28.49 ± 0.00		
3.104	+3.34 ± 0.02	Sealed in Ag tube	−28.51 ± 0.02	−40.69 ± 0.02	−42.26 ± 0.02
		Weighed in tin cup	−28.52 ± 0.01		
4.598	+3.32 ± 0.03	Sealed in Ag tube	−28.60 ± 0.02	−40.78 ± 0.02	−42.24 ± 0.02
		Weighed in tin cup	−28.59 ± 0.02		
		Average			−42.25 ± 0.01 (n = 4)

**FIGURE 4:**
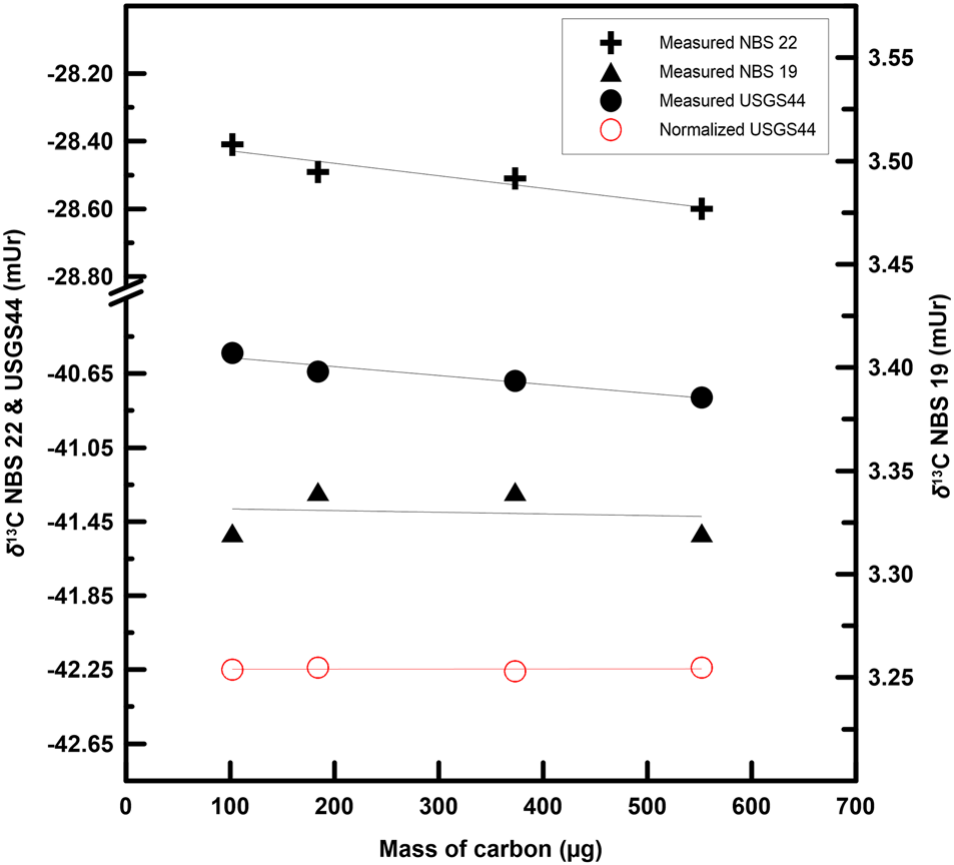
Measured *δ* ^13^C values and normalized *δ* ^13^C_VPDB_ values of USGS44 as a function of mass. Each data point represents three analyses by the RSIL, except for NBS 22, where each data point includes six analyses with three samples in silver tubes and three samples in tin capsules. All data were produced during one analytical sequence

The measured *δ* ^13^C values of NBS 22 and USGS44 drifted in the same direction, 0.19 mUr and 0.24 mUr, respectively, when the sample mass was changed from 0.85 mg to 4.6 mg (Figure 4). However, the measured *δ* ^13^C values of NBS 19 appeared to change little, and the normalized *δ* ^13^C_VPDB_ values of USGS44 are consistent within each mass‐amount group.

The average *δ* ^13^C_VPDB_ value of −42.25 ± 0.01 mUr (Table 6) for USGS44 obtained from four different sample amounts show that calcium carbonate and NBS 22 oil reacted similarly with sample masses ranging between 102 μg and 552 μg as carbon. Similar tests were also carried out at BGC‐IsoLab with a total of 66 aliquots of USGS44 with masses ranging from 0.5 mg to 7.5 mg; the overall standard deviation was 0.05 mUr. These observations give confidence in the approach used in determination of the final *δ* ^13^C_VPDB_ values of USGS44. However, these observations may not apply to other materials with a different chemical matrix. A careful evaluation of sample matrix effects is always recommended when RM calibration work is performed.

### 3.5 The *δ* ^13^C_VPDB_ values from EA/IRMS measurements

Three sets of EA data were produced in this study. Dataset 1: the *δ* ^13^C_VPDB_ value of USGS44 was determined from analysis of USGS44, NBS 19, and LSVEC and normalized to a LSVEC *δ* ^13^C_VPDB_ value of −46.6 mUr exactly (two‐point normalization). Dataset 2, the *δ* ^13^C_VPDB_ value of USGS44 was obtained directly against LSVEC with a *δ* ^13^C_VPDB_ value of −46.6 mUr exactly (one‐point normalization). The computation of the final *δ* ^13^C_VPDB_ value and uncertainty presented in Table 7 was performed using the Monte Carlo method^47^ as described in a similar application,^48^, ^50^ using codes written in the OpenBUGS software^49^ (Appendix A, supporting information). This method fully accounts for the uncertainty in the measurement of USGS44, the uncertainty in the measurement of the two RMs, and the uncertainty of the accepted delta values of the RMs. Lastly, the total of eight *δ* ^13^C_VPDB‐LSVEC_ values of the three laboratories were combined to obtain a consensus using a multivariate Gaussian meta‐analysis model^48^, ^50^ programed in OpenBUGS (Appendix B, supporting information). The resulting *δ* ^13^C_VPDB‐LSVEC_ value of USGS44 is −42.210 with a combined standard uncertainty of 0.048 mUr and a 95% uncertainty interval of [−42.31 mUr, −42.11 mUr]. A consistent average value was observed from both two‐point normalization and one‐point normalization. This indicates that the scale correction is insignificant (<0.01 mUr), whether using two‐point normalization or one‐point normalization when the unknown sample (USGS44) has a *δ* ^13^C value very close to that of the anchor RM (LSVEC). The high‐precision measurements carried out by three laboratories yielded a consistent difference in the *δ* ^13^C_VPDB_ value of −4.390 ± 0.071 mUr between LSVEC and USGS44 from data in Table 7. Table 8 summarizes the third dataset of *δ* ^13^C_VPDB‐LSVEC_ values that were obtained from analysis of USGS44, NBS 19, and NBS 22 by EA/IRMS and normalized to a NBS 22 *δ* ^13^C_VPDB_ value of −30.03 mUr (two‐point normalization). Surprisingly, the average *δ* ^13^C_VPDB_ value of −42.268 ± 0.069 mUr for USGS44 from column 5 of Table 8, in which NBS 22 (*δ* ^13^C_VPDB_ value = −30.03 mUr) was used as an anchor point, does not agree with the value of −42.210 ± 0.048 mUr from column 4 of Table 7, where LSVEC was used as an anchor point. For most routine measurements of *δ* ^13^C_VPDB_, a difference of 0.058 mUr between different runs is acceptable, based on 0.06 mUr acceptance criterion for repeatability of *δ* ^13^C measurements of modern IRMS instruments. However, for this work, substantial effort was made to achieve a higher precision. This 0.058 mUr discrepancy deserves some discussion: (1) Appropriate choice of RMs is crucial as discussed by Meier‐Augenstein and Schimmelmann.^51^ The narrower the *δ* value range covered by the RMs, the less accurate the resulting normalized measurements of samples with *δ* values outside that bracket. However, we do not believe that this is the case in this study because stringent carbon blank corrections were applied in all measurements in the three laboratories, and the quantitative conversion was carefully evaluated, and these are the two most dominant factors that contribute to less accurate normalized *δ* values outside that RM's bracket, (2) Could the issue be either that the value of −30.03 mUr for NBS 22 was incorrect in 2006 or that the LSVEC used in that work had been compromised? If the latter were true, the USGS44 value from Table 7 would have been more positive than the value of −42.210 mUr, which would make the discrepancy in USGS44 values even larger between the values from Table 7 and Table 8. If one uses a value of USGS44 = −42.210 mUr obtained from Table 7 to re‐normalize the NBS 22 values in column 4 of Table 8, a set of new values for NBS 22 can be calculated (see last column of Table 8). The resulting *δ* ^13^C_VPDB‐LSVEC_ value of NBS 22 is –29.988 mUr with a combined standard uncertainty of 0.054 mUr and a 95% uncertainty interval of [−30.10 mUr, −29.88 mUr]. The computation of the final *δ* ^13^C_VPDB‐LSVEC_ value of NBS 22 and the uncertainty presented in Table 8 (Appendix C and Appendix D, supporting information) was performed using the same approach as described above for Table 7. Further discussion about NBS 22 can be found in section 3.7.

**TABLE 7:** The *δ* ^13^C_VPDB_ values of USGS44 measured by EA/IRMS and normalized to the *δ* ^13^C_VPDB_ value of LSVEC = −46.6 mUr exactly. [n.d. = not determined]

Description	*δ* ^13^C_VPDB_ of NBS 19 CaCO_3_ (mUr)	*δ* ^13^C_VPDB_ of LSVEC Li_2_CO_3_ (mUr)	*δ* ^13^C_VPDB_ of USGS44 CaCO_3_ (mUr)	Difference between LSVEC and USGS44
RSIL 1	+1.95 ± 0.03 (n = 8)	−46.60 ± 0.04 (n = 8)	−42.206 ± 0.040	−4.394 ± 0.081
RSIL 2	+1.95 ± 0.03 (n = 8)	−46.60 ± 0.04 (n = 8)	−42.221 ± 0.040	−4.379 ± 0.079
BGC‐IsoLab	+1.95 ± 0.03 (n = 8)	−46.60 ± 0.02 (n = 31)	−42.166 ± 0.023	−4.434 ± 0.039
Geotop 1	+1.95 ± 0.05 (n = 3)	−46.60 ± 0.02 (n = 5)	−42.185 ± 0.026	−4.415 ± 0.055
Geotop 2	+1.95 ± 0.03 (n = 3)	−46.60 ± 0.02 (n = 9)	−42.297 ± 0.032	−4.303 ± 0.060
RSIL 3	n.d.	−46.60 ± 0.02 (n = 15)	−42.202 ± 0.022	−4.398 ± 0.038
RSIL 4	n.d.	−46.60 ± 0.02 (n = 19)	−42.195 ± 0.019	−4.405 ± 0.036
RSIL 5	n.d.	−46.60 ± 0.04 (n = 11)	−42.210 ± 0.041	−4.389 ± 0.083
		Consensus^a^	−42.210 ± 0.048	−4.390 ± 0.071

^**a**^ The consensus value is based on the eight individual values and standard uncertainties given in column 4. It is calculated using the NIST Consensus Builder (Linear Pool option).^46^ The ± 0.04 is the standard uncertainty of the consensus and can be expanded by multiplication by 2 to obtain the 95% uncertainty band.

**TABLE 8:** The *δ* ^13^C_VPDB‐LSVEC_ values of NBS 22 measured by EA/IRMS and normalized to the *δ* ^13^C_VPDB‐LSVEC_ value of USGS44 = −42.21 mUr. [n.d. = not determined]

Description	Standard deviation of NBS 19 CaCO_3_ (mUr)	*δ* ^13^C_VPDB_ of IAEA‐603 CaCO_3_ (mUr)		Standard deviation of NBS 22 oil (mUr)	*δ* ^13^C_VPDB_ of USGS44 CaCO_3_ NBS 22 = −30.03 (mUr)	*δ* ^13^C_VPDB_ of NBS 22 oil USGS44 = −42.21 (mUr)
BGC‐IsoLab 1^a^	n.d	+2.46 ± 0.03 (n = 10)		0.03 (n = 10)	−42.260 ± 0.082	−29.997 ± 0.056
BGC‐IsoLab 2^a^	n.d	+2.46 ± 0.04 (n = 10)		0.02 (n = 10)	−42.328 ± 0.079	−29.952 ± 0.055
BGC‐IsoLab 3^a^	n.d	+2.46 ± 0.01 (n = 10)		0.02 (n = 10)	−42.318 ± 0.077	−29.959 ± 0.054
BGC‐IsoLab 4^a^	n.d	+2.46 ± 0.02 (n = 10)		0.01 (n = 10)	−42.276 ± 0.075	−29.986 ± 0.052
BGC‐IsoLab 5^a^	n.d	+2.46 ± 0.03 (n = 8)		0.02 (n = 9)	−42.317 ± 0.075	−29.959 ± 0.052
BGC‐IsoLab 6^a^	n.d	+2.46 ± 0.02 (n = 15)		0.01 (n = 10)	−42.324 ± 0.074	−29.955 ± 0.051
BGC‐IsoLab 7^a^	n.d	+2.46 ± 0.02 (n = 10)		0.01 (n = 10)	−42.187 ± 0.073	−30.045 ± 0.052
BGC‐IsoLab 8^a^	n.d	+2.46 ± 0.02 (n = 10)		0.01 (n = 10)	−42.211 ± 0.073	−30.030 ± 0.051
BGC‐IsoLab 9^b^	0.03 (n = 9)	n.d		0.01 (n = 15)	−42.366 ± 0.076	−29.928 ± 0.055
BGC‐IsoLab 10^b^	0.03 (n = 9)	n.d		0.01 (n = 15)	−42.309 ± 0.073	−29.964 ± 0.054
RSIL 1^b^	0.02 (n = 8)	+2.48 ± 0.02 (n = 6) ^c^		0.02 (n = 10)	−42.248 ± 0.076	−30.005 ± 0.057
RSIL 2^b^	0.01 (n = 6)	+2.44 ± 0.04 (n = 6) ^c^		0.01 (n = 13)	−42.278 ± 0.075	−29.985 ± 0.055
RSIL 3^b^	0.01 (n = 6)	+2.45 ± 0.03 (n = 6) ^c^		0.01 (n = 9)	−42.268 ± 0.073	−29.992 ± 0.054
RSIL 4^b^	0.05 (n = 3)	n.d		0.01 (n = 6)	−42.252 ± 0.124	−30.004 ± 0.091
RSIL 5^b^	0.05 (n = 3)	n.d		0.01 (n = 6)	−42.241 ± 0.093	−30.009 ± 0.068
RSIL 6^b^	0.02 (n = 3)	n.d		0.01 (n = 6)	−42.258 ± 0.105	−29.998 ± 0.063
RSIL 7^b^	0.03 (n = 3)	n.d		0.01 (n = 6)	−42.239 ± 0.079	−30.011 ± 0.059
RSIL 8^b^	0.03 (n = 10)	+2.49 ± 0.01 (n = 10) ^c^		0.01 (n = 15)	−42.255 ± 0.072	−30.000 ± 0.054
Consensus^d^					−42.268 ± 0.069	−29.988 ± 0.054^d^

^a^ Value was determined using NBS 22 oil^10^, ^11^ and +2.46 mUr for IAEA‐603 calcium carbonate.^2^, ^3^

^b^ Value was determined using NBS 22 oil^10^, ^11^ and +1.95 mUr for NBS 19 calcium carbonate.

^c^ IAEA‐603 was not used as anchor point. Value was determined by assigning a value of −30.03 mUr for NBS 22 oil^10^, ^11^ and +1.95 mUr for NBS 19 calcium carbonate.

^**d**^ Consensus value for NBS 22 is based on the 18 individual values and standard uncertainties given in columns 5 and 6, and correlations given in Appendix B (supporting information). They are calculated using the methods of Meija and Chartrand^50^ via Monte Carlo analysis implemented in OpenBUGS.^49^ The codes are given in Appendix A (supporting information). The ± 0.069 (and ± 0.054) are standard uncertainties and can be expanded by multiplication by 2 to obtain 95% uncertainty bands.

A concern about the impact of incorrectly assigning the *δ* ^18^O value of the reference injection gas in Isodat arose during the project. Does it make any difference whether the reference injection gas is assigned as 0 mUr or +23 mUr (or −23 mUr)? We confirmed that as long as one normalizes the *δ* ^13^C measurements with two anchors, the impact upon the normalized *δ* ^13^C_VPDB_ value of the assigned *δ* ^18^O of reference injection CO_2_ is insignificant (<0.01 mUr).

### 3.6 The *δ* ^13^C_VPDB_ values from dual‐inlet measurements

**FIGURE 5:**
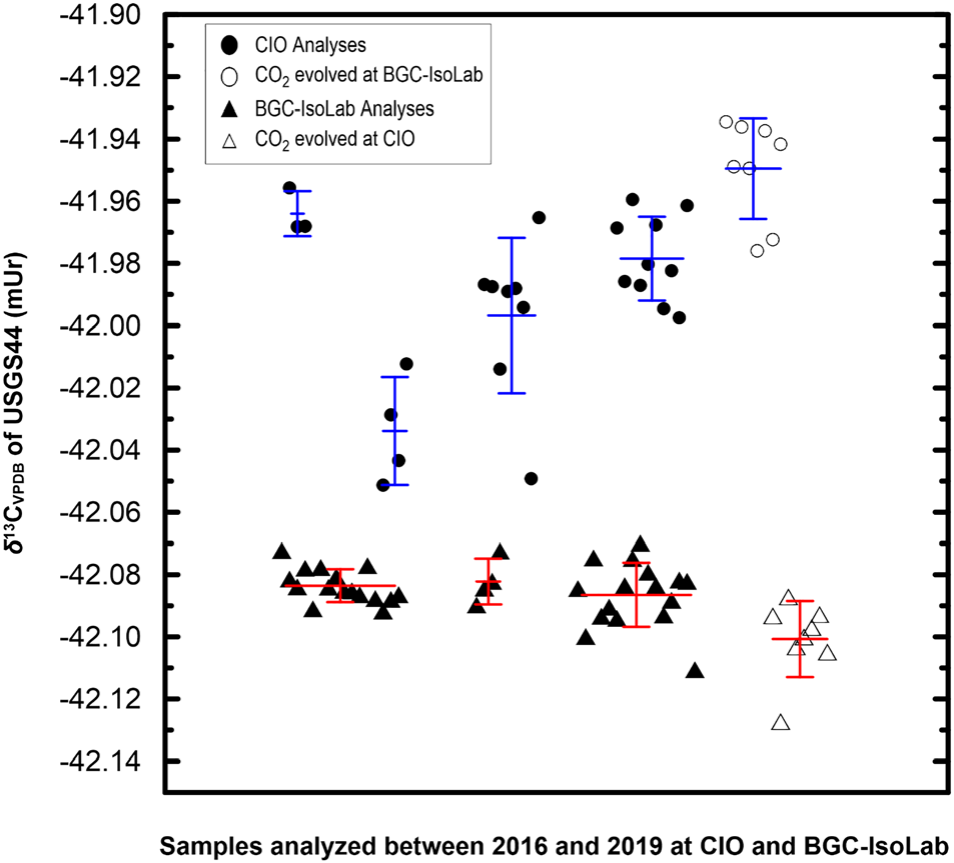
Individual DI‐IRMS *δ* ^13^C_VPDB_ measurements of USGS44 by BGC‐IsoLab and CIO between 2016 and 2019. Values are corrected for cross contamination

Ideally, an accurate *δ* ^13^C_VPDB_ determination of USGS44 should have been carried out with two‐point normalization. However, a second scale anchor that is independent of LSVEC currently does not exist. To overcome this deficiency, the best method to obtain an accurate *δ* ^13^C measurement with only one RM, NBS 19 or IAEA‐603, is that described by Meijer et al^34^ and Meijer^37^ in which the cross‐contamination needs to be carefully evaluated and corrected. At BGC‐IsoLab, CO_2_ from four aliquots of USGS44 was extracted during three time periods between August 2016 and April 2018 (Table 9 and Figure 5). A total of six CO_2_ samples was produced from each aliquot and analyzed on a MAT 253 isotope‐ratio mass spectrometer. The CO_2_ measurements from 2016 and 2017 were normalized against CO_2_ evolved from NBS 19, while measurements of the CO_2_ evolved from USGS44 in 2018 were normalized against IAEA‐603.^2^, ^3^ To confirm comparability of CO_2_ sample preparation between the CIO and BGC‐IsoLab facilities, CIO prepared CO_2_ from eight USGS44 and three NBS 19 samples. These samples were analyzed by BGC‐IsoLab (Table 9 and Figure 5). Table 9 summarizes all measured values of USGS44, NBS 19, and IAEA‐603. Individual results are displayed in Figure 5. The weighted mean *δ* ^13^C_VPDB_ value of 36 measurements made at BGC‐IsoLab is −42.0847 mUr, which we round to −42.08 mUr for use in normalization (column 4 of Table 1; discussed below) and to −42.085 mUr with a standard deviation of 0.008 mUr in Table 9. To minimize scale contraction effects, BGC‐IsoLab studied the value of *η* for the MAT253 on two separate occasions by conducting idle time experiments (Figure 2). The experiments reveal that the MAT 253 does not suffer from a measurable cross‐contamination value when an idle time of 60 s or longer is chosen. However, because such long idle times are not practical during routine analyses, as they dramatically reduce sample throughput, an idle time of 15 s was selected for all measurements. The experiments further showed that the value of *η* is very stable over time, and the uncertainty that it introduces is within the measurement uncertainty. Considering a *δ* ^13^C span of 44.03 mUr, which is the difference between the *δ* ^13^C_VPDB_ values of NBS 19 and our measurements of USGS44, the uncertainty introduced by cross contamination (taking the average and standard deviation of *η* values determined for *δ* ^13^C_VPDB_ measurements) is less than 0.006 mUr. The USGS44 CO_2_ gases produced in 2016 and 2017 were analyzed on the MAT 252 isotope‐ratio mass spectrometer at BGC‐IsoLab that is known to have minimal cross contamination^8^ to verify the validity of the applied *η* correction on the MAT 253 system. Standardization was achieved using the eight CO_2_ gases evolved from NBS 19 syntheses from 2016. The average *δ* ^13^C_VPDB_ and *δ* ^18^O_VPDB_ values and standard deviations of the MAT252 measurements were −42.08 ± 0.01 mUr and −15.75 ± 0.07 mUr, respectively. Within analytical uncertainty, the MAT 253 and MAT 252 isotope‐ratio mass spectrometers produce identical *δ* ^13^Cvalues for CO_2_ evolved from USGS44, thus supporting our contention that the applied corrections to the MAT 253 measurements are valid.

**TABLE 9:** Scale‐normalized *δ* ^13^C_VPDB_ and *δ* ^18^O_VPDB_ values of RMs of offline DI‐IRMS measurements in this study. [Uncertainties are 1‐σ uncertainties. The *δ* ^18^O_VPDB_ values are for information only because USGS44 is not suitable as a *δ* ^18^O reference material due to its small grain size. USGS44‐CIO and USGS44‐BGC‐IsoLab CO_2_ gases were prepared, respectively, by CIO and BGC‐IsoLab and are not included in the values for averages]

Laboratory	CO_2_ production period	Reference material	*δ* ^13^C_VPDB_ (mUr)	*δ* ^18^O_VPDB_ (mUr)	Number of syntheses
BGC‐IsoLab	August–December 2016	USGS44 #1	−42.079 ± 0.013	−15.793 ± 0.023	4
		USGS44 #2	−42.083 ± 0.013	−15.801 ± 0.020	4
		USGS44 #3	−42.083 ± 0.013	−15.818 ± 0.021	4
		USGS44 #4	−42.088 ± 0.013	−15.823 ± 0.021	4
		NBS 19	+1.950 ± 0.014	−2.200 ± 0.020	8
	March–June 2017	USGS44 #1	−42.090 ± 0.019	−15.735 ± 0.015	1
		USGS44 #2	−42.084 ± 0.015	−15.767 ± 0.027	1
		USGS44 #3	−42.082 ± 0.011	−15.754 ± 0.019	1
		USGS44 #4	−42.072 ± 0.013	−15.746 ± 0.018	1
		NBS 19	+1.950 ± 0.012	−2.200 ± 0.018	8
	January–April 2018	USGS44 #1	−42.088 ± 0.015	−15.725 ± 0.018	4
		USGS44 #2	−42.086 ± 0.013	−15.701 ± 0.020	4
		USGS44 #3	−42.081 ± 0.014	−15.636 ± 0.018	4
		USGS44 #4	−42.091 ± 0.013	−15.708 ± 0.023	4
		IAEA‐603	+2.460 ± 0.013	−2.370 ± 0.019	10
	July 2019	USGS44‐CIO	−42.101 ± 0.012	−15.702 ± 0.045	8
		NBS 19	+1.950 ± 0.003	−2.200 ± 0.042	3
	Average	USGS44	−42.085 ± 0.008	−15.751 ± 0.070	36
CIO	November 2016	USGS44 #1	−41.964 ± 0.007	−15.709 ± 0.048	3
		NBS 19	+1.950 ± 0.017	−2.200 ± 0.014	3
	April 2017	USGS44 #1	−42.034 ± 0.017	−15.646 ± 0.018	4
		NBS 19	+1.950 ± 0.000	−2.200 ± 0.055	2
		LSVEC	−46.464 ± 0.046	−26.507 ± 0.137	4
	March 2018	USGS44 #1	−41.994 ± 0.013	−15.642 ± 0.026	4
		NBS 19	+1.950 ± 0.018	−2.200 ± 0.023	3
		LSVEC	−46.292 ± 0.051	−26.730 ± 0.037	4
		USGS44 #2	−42.000 ± 0.036	−15.662 ± 0.036	4
		IAEA‐603	+2.460 ± 0.023	−2.370 ± 0.038	3
	May 2019	USGS44 #1	−41.978 ± 0.013	−15.646 ± 0.048	10
		USGS44‐BGC‐IsoLab	−41.950 ± 0.016	−15.655 ± 0.030	8
		IAEA‐603	+2.481 ± 0.015	−2.381 ± 0.042	5
		NBS 19	+1.950 ± 0.018	−2.200 ± 0.033	9
	Average	USGS44	−41.992 ± 0.022	−15.657 ± 0.022	25

At CIO, CO_2_ was evolved from three USGS44 samples in 2016, four in 2017, eight in 2018, and ten in 2019 (Table 9). A one‐point normalization was performed by analysis of CO_2_ evolved from NBS 19 or IAEA‐603. The weighted mean *δ* ^13^C_VPDB_ value and standard deviation for USGS44 from these 25 cross‐contamination‐corrected measurements are −41.992 ± 0.022 mUr (Table 9) (the uncertainty in the mean being a factor of 5 smaller but deemed not realistic due to the contribution of systematic biases). For *δ* ^18^O_VPDB_ measurements, the values are −15.657 ± 0.022 mUr (Table 9). The individual *δ* ^13^C_VPDB_ measurements are shown in Figure 5, where the significant variation of the mean values over the years can be seen. This variation points to the limitation of the accuracy achieved for the correction for the various scale contraction contributions that are mentioned above. Table 9 summarizes all CIO‐measured values of USGS44, NBS 19, and IAEA‐603. The weighted mean *δ* ^13^C_VPDB_ value of 25 measurements made at CIO is rounded to −41.99 mUr and is used in normalization (column 5 of Table 1; discussed below).

The cross‐contamination‐corrected, mean‐weighted *δ* ^13^C_VPDB_ values determined by BGC‐IsoLab and CIO, respectively, −42.08 ± 0.01 mUr and −41.99 ± 0.02 mUr, are not as identical within analytical uncertainty as one might have expected (Table 9 and Figure 5). Rather, they differ by 0.093 mUr, which is substantially in excess of the standard deviations of BGC‐IsoLab and CIO of 0.008 and 0.022 mUr, respectively. The results from CIO are significantly less negative than those from BGC‐IsoLab. This is true for both *δ* ^13^C and *δ* ^18^O measurements. To rule out possible systematic differences due to the CO_2_ production from carbonates, in 2019 CIO analyzed CO_2_ evolved from eight USGS44 and five IAEA‐603 samples prepared by BGC‐IsoLab. Likewise, BGC‐IsoLab analyzed CO_2_ produced at CIO in the form of eight CO_2_ samples evolved from USGS44 and three evolved from NBS 19 (Table 9 and Figure 5). At BGC‐IsoLab, the *δ* ^13^C_VPDB_ value of the CO_2_ prepared by CIO from USGS44 is more negative by 0.016 mUr than their average value of −42.085 ± 0.008 mUr (Table 9). At CIO, the *δ* ^13^C_VPDB_ value of the CO_2_ prepared by BGC‐IsoLab from USGS44 is more positive by 0.042 mUr than their average value of −41.992 ± 0.022 mUr (Table 9). The cause of the differences among these values is unknown. We conclude that the *δ* ^13^C_VPDB_ and *δ* ^18^O_VPDB_ differences between CIO and BGC‐IsoLab are not caused by the carbonate treatment to generate CO_2_ but suggest a scale realization or instrument problem. As a rule of thumb, the more stretched scale, and thus the one producing more negative results for materials like USGS44, is more likely to be the right one, but that is only true for scales prior to correction. Obviously, there is always the possibility that one stretches the scale by too much. In this case, both groups have gone to considerable lengths to try to produce an isotope‐delta scale in which a milliurey (‰) truly represents a milliurey, but we are confronted with a difference that we deem beyond our estimated scale realization uncertainty. In Figure 5, it is clear that BGC‐IsoLab has been able to perform scale contraction correction with higher precision than CIO over the years, which, however, does not necessarily imply that that correction is more accurate.

### 3.7 Comparison of *δ* ^13^C_VPDB_ measurements by DI‐IRMS and EA/IRMS

In this study, the EA/IRMS measurements of USGS44 give a *δ* ^13^C_VPDB_ value of −42.21 ± 0.05 mUr and DI‐IRMS values of −42.08 ± 0.01 mUr from BGC‐IsoLab and −41.99 ± 0.02 mUr from CIO. This relatively large 0.13‐mUr (or 0.22‐mUr) difference between the two techniques, and the difference of 0.09 mUr within DI‐IRMS values, merit discussion. We identify three possible causes for the observed difference between the EA/ and DI‐IRMS measurement results: (a) the EA/IRMS measurements are faulty, (b) the DI‐IRMS measurements are problematic, or (c) the scaling of the *δ* ^13^C_VPDB‐LSVEC_ scale is incorrect. The EA/IRMS measurements were conducted at three different laboratories using different setups and yet provide USGS44 *δ* ^13^C_VPDB‐LSVEC_ values with a 95% uncertainty interval of 0.05 mUr (see supporting information). The very high precision that was achieved suggests that the analytical setup in three laboratories, along with the relevant off‐ and online corrections, are correct. CO_2_ evolved at both CIO and BGC‐IsoLab and analyzed by the other yielded *δ* ^13^C_VPDB_ results compared with locally evolved CO_2_, which indicates that production of CO_2_ is not an issue. If the differences between locally evolved CO_2_ and CO_2_ from the other laboratory were significant, it would make the difference between the two laboratories even larger. The BGC‐IsoLab DI‐IRMS measurements were conducted on two different instruments over a period of several years that both provided the same value within analytical uncertainty. This suggests that the DI‐IRMS analytical instrumentation and relevant corrections by BGC‐IsoLab are correct or that unrecognized bias affects the MAT 252 and MAT 253 instruments approximately equally.

Although LSVEC is unsuitable as a second scale anchor for the *δ* ^13^C_VPDB‐LSVEC_ scale as its *δ* ^13^Cvalue increases due to its gradual reaction with atmospheric CO_2_, we would like to point out that if well‐preserved LSVEC is used with the EA method, reproducible *δ* ^13^C measurements can be achieved, as was demonstrated in measurements on USGS40 and USGS41 carried out in 2003,^26^ measurements on USGS41a in 2016,^14^ and in this work. Nevertheless, to ensure that some of the measurements were independent from LSVEC, NBS 22 was also selected in this project as a scale anchor because of its stable nature. The NBS 22 value of −30.03 mUr is based on an LSVEC value of −46.6 mUr exactly,^10^, ^11^ but the problematic nature of LSVEC calls into question values which were based on its changing *δ* ^13^C value. Evaluating the discrepancy of 0.058 mUr determined for USGS44 between the EA value of −42.210 ± 0.048 mUr from Table 7 and the value of −42.268 ± 0.069 mUr from Table 8, we suspected that the determination of NBS 22 at that time was slightly flawed, perhaps by the use of LSVEC that had been exposed to atmospheric moisture because the importance of using pristine LSVEC had not been recognized in 2006. A slight shift in the LSVEC value towards a more positive *δ* ^13^C value could result in a value for NBS 22 being too negative. The re‐normalized value of −29.99 ± 0.05 mUr of NBS 22 from Table 8 using a value of −42.21 ± 0.05 mUr of USGS44 from Table 7 is identical to the value in Table 2 of Qi et al^14^ where a value of −46.6 mUr was used for LSVEC, and identical to the value in Table 1 of Qi et al^26^ (NBS 22 = −29.91 mUr when LSVEC = −46.48 mUr, NBS 22 = −29.99 mUr when LSVEC = −46.60 mUr).

The *δ* ^13^C_VPDB_ values of selected RMs normalized to BGC‐IsoLab and CIO *δ* ^13^C_VPDB_ values of −42.08 and −41.99 mUr for USGS44, respectively, are shown in columns 4 and 5 of Table 1. It is instructive to compare the normalized *δ* ^13^C_VPDB_ values of some selected RMs (such as NBS 22, USGS40, and LSVEC) in columns 4 and 5 with previous measurements. The normalized *δ* ^13^C_VPDB_ values of NBS 22 and LSVEC using the BGC‐IsoLab value of −42.08 mUr for USGS44 are −29.90 mUr and −46.46 mUr, respectively. These two values are in excellent agreement with the *δ* ^13^C_VPDB_ value of Qi et al^26^ of NBS 22 of −29.91 ± 0.03 mUr, published with an assumed LSVEC *δ* ^13^C_VPDB_ value of −46.48 mUr. Likewise, the normalized *δ* ^13^C_VPDB_ value of −26.23 mUr of USGS40 l‐glutamic acid with the BGC‐IsoLab USGS44 value agree very well with the value of −26.24 mUr of USGS40 from Qi et al.^26^ With the same comparison by normalizing to CIO *δ* ^13^C_VPDB_ values of −41.99 mUr for USGS44, values of −29.83 mUr and −46.36 mUr were obtained for NBS 22 and LSVEC, respectively. These values are in fair agreement with the *δ* ^13^C_VPDB_ value of Qi et al^26^ of NBS 22 of −29.91 ± 0.03 mUr, published with an assumed *δ* ^13^C_VPDB_ value of LSVEC of −46.48 mUr. The normalized value of −46.36 mUr for LSVEC is between the *δ* ^13^C_VPDB_ values of LSVEC (−46.25 and −46.84 mUr) reported by Verkouteren and Klinedinst.^9^ The excellent agreement in *δ* ^13^C_VPDB_ values of USGS44 between the DI‐IRMS and EA measurements suggests that: (a) the EA/IRMS measurements are not faulty and (b) the discrepancy in *δ* ^13^C_VPDB_ values between the EA and DI‐IRMS measurements is caused by scaling due to an incorrect value of −46.6 mUr assigned to LSVEC. If a *δ* ^13^C_VPDB_ value of −46.46 mUr (BGC‐IsoLab DI value, Table 1) were used for normalizing data in Table 7, a *δ* ^13^C_VPDB‐LSVEC_ value of −42.08 mUr for USGS44 would have been obtained.

If the “true” value of LSVEC is nearer −46.48 mUr than the 2006 value of −46.60 mUr,^10^, ^11^ this suggests that the measurement of LSVEC by Ghosh et al^8^ of −46.607 ± 0.057 mUr suffered from non‐quantitative extraction of CO_2_ from LSVEC. Verkouteren and Klinedinst^9^ list LSVEC values from seven laboratories that vary between −46.25 and −46.84 mUr (standard deviation: 0.17 mUr). At the time, it was thought that the large differences among the values resulted from different scale contraction effects in the selected laboratories. Inconsistent H_3_PO_4_ digestion of LSVEC probably added to the variations reported in 2004,^9^ and this subject remains to be investigated using LSVEC and other high‐purity lithium carbonates. The wide variation in *δ* ^13^C_VPDB_ values of LSVEC reported by Verkouteren and Klinedinst^9^ supports the contention that it is difficult to extract carbon quantitatively and measure the *δ* ^13^C value of LSVEC reproducibly with high accuracy in multiple laboratories. In retrospect, LSVEC was a poor choice for a scale anchor.

These findings highlight several points which need to be discussed. First, the introduction of LSVEC and adoption of its *δ* ^13^C_VPDB_ value of −46.6 mUr exactly as a second scale anchor have probably caused users to overestimate the scale compression of their isotope‐ratio mass spectrometers. Second, and more importantly, the isotope geochemistry community may want to consider whether:

1. LSVEC with a consensus value of −46.6 mUr is retained as a second anchor (even though the results herein indicate that its consensus value is not correct), with this scale realized using secondary RMs such as NBS 22 or USGS44, in an identical fashion to realization of the *δ* ^2^H_VSMOW‐SLAP_ and *δ* ^18^O_VSMOW‐SLAP_ scales,^52^ or
2. LSVEC is replaced as the second anchor by another RM with adoption of a new VPDB_202X scale.

For now, we recommend the continued use of the VPDB‐LSVEC scale until USGS44 or another suitable second scale anchor for the *δ* ^13^C_VPDB_ scale has been accepted by the CIAAW and IAEA experts' panel.

The two high‐accuracy DI‐IRMS measurements reported in this study by BGC‐IsoLab of −42.08 ± 0.01 mUr (combined standard uncertainty) and CIO of −41.99 ± 0.02 mUr (combined standard uncertainty) do not agree even by expanding their uncertainties with a coverage factor (*k*) of 2. Therefore, it is unadvisable to combine them to recommend an average or weighted average DI‐IRMS *δ* ^13^C_VPDB_ value for USGS44. Additional DI‐IRMS *δ* ^13^C_VPDB_ measurements are needed to solve this conundrum. With the knowledge that we have now, the value of −42.08 mUr for USGS44 is preferred because: (1) the *δ* ^13^C_VPDB_ value of −42.08 mUr from BGC‐IsoLab was determined on two different instruments over a period of several years that both provided the same value, and its uncertainty of 0.01 mUr is better than that of −41.99 ± 0.02 mUr from CIO; (2) the re‐normalized *δ* ^13^C_VPDB_ value of −46.46 mUr for LSVEC using the value of −42.08 from BGC‐Isolab agrees well with the value in Table 1 (In 1995, Stichler^6^ reported a value of −46.48 ± 0.15 mUr); (3) the re‐normalized value of −29.90 ± 0.05 mUr for NBS 22 using BGC‐IsoLab's USGS44 value is in excellent agreement with the *δ* ^13^C_VPDB_ value of Qi et al^26^ (2003) of NBS 22 of −29.91 ± 0.03 mUr, published with an assumed *δ* ^13^C_VPDB_ value of LSVEC of −46.48 mUr. Likewise, if one re‐normalizes the NBS 22 value of −29.99 ± 0.07 mUr published in Table 2 of Qi et al^14^ (2016) to a *δ* ^13^C_VPDB_ value of LSVEC of −46.48 mUr, the re‐normalized value is −29.91 ± 0.07 mUr, which is in excellent agreement with the re‐normalized value of −29.90 ± 0.05 mUr.

A comparison of the normalized *δ* ^13^C_VPDB_ values of USGS44 obtained by EA (Table 3) and by GasBench/MultiCarb analysis (Table 4) results in an interesting observation. Although the *δ* ^13^C_VPDB_ values in Tables 3 and 4 were normalized to NBS 19, the average *δ* ^13^C_VPDB_ value for USGS44 of −40.08 ± 0.07 mUr from EA measurement is 1.86 mUr more positive than that of −41.94 ± 0.22 mUr from GasBench/MultiCarb measurements. The value from the GasBench/MultiCarb analysis is nearer the average values reported in Table 9 of −42.085 ± 0.008 and −41.992 ± 0.022 mUr determined by DI‐IRMS measurements. This indicates the importance of correcting for the carbon blank and of using two‐point normalization for the stable carbon isotope‐delta scale when an EA is used to combust samples. The *δ* ^13^C scale contraction is substantially less when analyzing carbonates with a GasBench or MultiCarb system.

## 4 SUMMARY AND CONCLUSIONS

The new high‐purity, well‐homogenized calcium carbonate *δ* ^13^C reference material USGS44 is stable and has a *δ* ^13^C_VPDB‐LSVEC_ two‐anchor value of −42.21 ± 0.05 mUr (combined standard uncertainty) obtained by EA/IRMS, a value that is recommended for the *δ* ^13^C measurements both by EA/IRMS and by DI‐IRMS. A *δ* ^13^C_VPDB‐LSVEC_ value of −29.99 ± 0.05 mUr of NBS 22 oil is recommended. Both the above *δ* ^13^C_VPDB‐LSVEC_ values are henceforth the recommended values of these secondary isotopic reference materials on the VPDB‐LSVEC scale having an assigned *δ* ^13^C value for NBS 19 of +1.95 mUr exactly and a *δ* ^13^C value of −46.6 mUr exactly for LSVEC. A single‐anchor DI‐IRMS measurement of USGS44 yielded a *δ* ^13^C_VPDB_ value of −42.08 ± 0.01 mUr (combined standard uncertainty) as measured on two different mass spectrometers over an interval of several years by the Max Planck Institute for Biogeochemistry, Jena, Germany (BGC‐IsoLab). A value of −41.99 ± 0.02 mUr (combined standard uncertainty) was measured by the Centre for Isotope Research (CIO), University of Groningen, Groningen, The Netherlands. These high accuracy DI‐IRMS measurements are not in agreement even with a coverage factor (*k*) of 2. Additional investigations are needed to solve this conundrum. Therefore, we recommend continued use of the two‐anchor VPDB‐LSVEC scale, but with realization of the *δ* ^13^C_VPDB‐LSVEC_ scale using RMs other than LSVEC Li_2_CO_3_, whose use is to be deprecated for *δ* ^13^C measurements. The discrepancy in *δ* ^13^C_VPDB_ values between EA measurement and DI‐IRMS is caused by scaling due to an incorrect value of −46.6 mUr assigned to LSVEC. If a *δ* ^13^C_VPDB_ value of −46.46 mUr were used for normalizing EA/IRMS data, a *δ* ^13^C_VPDB‐LSVEC_ value of −42.08 mUr for USGS44 would have been obtained, identical to the BGC‐IsoLab DI‐IRMS value. If the two‐anchor *δ* ^13^C_VPDB‐LSVEC_ scale is replaced by another scale, we recommend a *δ* ^13^C_VPDB_ value of −42.08 mUr for USGS44.

EA/IRMS *δ* ^13^C measurements can achieve high precision and high accuracy and EA values are comparable with the measurements by DI‐IRMS if two‐point normalization is applied using two appropriate reference materials with correctly assigned *δ* ^13^C_VPDB‐LSVEC_ values. As a carbonate relatively depleted in ^13^C, USGS44 is intended for daily use as a secondary isotopic reference material to normalize the *δ* ^13^C_VPDB‐LSVEC_ scale. It should be useful in quantifying drift with time,evaluating mass‐dependent isotopic fractionation (linearity correction), and adjusting isotope‐ratio‐scale contraction. Due to its fine grain size (smaller than 63 μm), it is not suitable as a *δ* ^18^O reference material.

USGS44 is available in units of 0.5 g in a glass vial; each vial is vacuum sealed in a plastic pouch. There is no limit on purchasing USGS44. It is available at http://isotopes.usgs.gov/lab/referencematerials.html.

5

### PEER REVIEW

The peer review history for this article is available at https://publons.com/publon/10.1002/rcm.9006.

## Supporting information


**Data S1** Supporting Information
