# Supplementary material for: USGS44, a new high‐purity calcium carbonate reference material for δ 13C measurements
Source: Rapid Commun Mass Spectrom. 2021 Jan 7;35(4):e9006. doi: 10.1002/rcm.9006 (PMC7816275; doi:10.1002/rcm.9006)
Supplement: Supplementary file 1 — Data S1 Supporting Information [file RCM-35-e9006-s001.docx]

Supplementary Materials for

**USGS44, a new high purity calcium carbonate reference material for *δ*^13^C measurements**

Haiping Qi^*^, Heiko Moossen, Harro A.J. Meijer, Tyler B. Coplen, Anita T. Aerts-Bijma, Lauren Reid, Heike Geilmann, Jürgen Richter, Michael Rothe, Willi A. Brand, Blaza Toman, Jacqueline Benefield, Jean-François Hélie

*Corresponding author. Email: haipingq@usgs.gov

**This file includes:**

Appendix A: Correlation matrices used to calculate consensus values for columns 5 and 6 of Table 8.

Appendix B: OpenBUGS codes for the 8 individual USGS44 v in Table 7**.**

Appendix C: OpenBUGS codes to obtain the 18 individual NBS22 values in Table 8.

Appendix D: Multivariate Gaussian meta-analysis model programed in OpenBUGS to obtain

the consensus USGS44 value for Table 8.

**Appendix A: Correlation matrices used to calculate consensus values for columns 5 and 6 of Table 8**

Correlation matrix for the 18 values as shown in column 5 of Table 8.


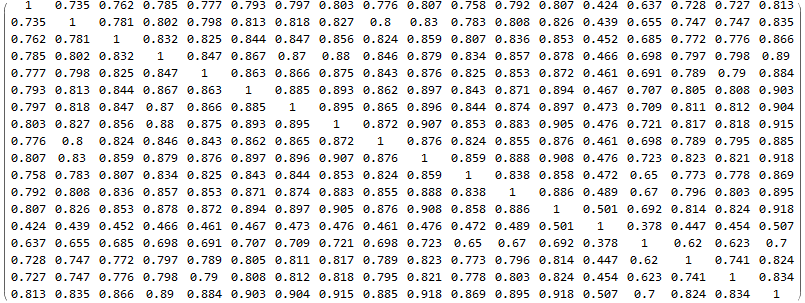


Correlation matrix for the 18 values as shown in column 6 of Table 8:


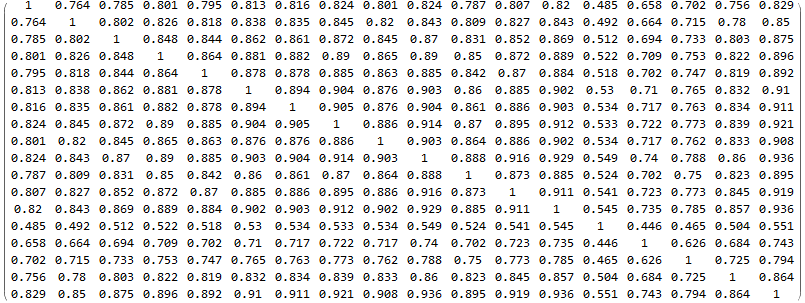


**Appendix B: OpenBUGS codes for the 8 individual USGS44 values in Table 7:**

{

NBS19<-1.95

LSVEC<--46.6

for( i in 1:5){

for(j in 1:3){matmr1[j,i]~dnorm(0,1.0E-5)

xins[j,i]~dnorm(0,0.0016)I(0.001,)

chsqns[j,i]~dgamma(0.5,0.5)

sigmr1[j,i]<-xins[j,i]/sqrt(chsqns[j,i])

}}

for( i in 1:173){measr1[i]~dnorm(matmr1[materialr1[i],dayr1[i]],sigmr1[materialr1[i],dayr1[i]])}

for(i in 1:2){coeffr1[i]<-(LSVEC-NBS19)/(matmr1[3,i]-matmr1[1,i])

additr1[i]<-LSVEC-coeffr1[i]*matmr1[3,i]

}

matmr1m<-(matmr1[1,1]+matmr1[1,2])/2

for(i in 3:5){coeffr1[i]<-(LSVEC-NBS19)/(matmr1[3,i]-matmr1m)

additr1[i]<-LSVEC-coeffr1[i]*matmr1[3,i]

}

for(i in 1:5){matnormr1[i]<-matmr1[2,i]*coeffr1[i]+additr1[i]

difr1[i]<-matmr1[3,i]-matnormr1[i]}

for(i in 1:3){matmb1[i]~dnorm(0,1.0E-5)

sigmb1[i]~dgamma(1.0E-5,1.0E-5)

}

for( i in 1:71){measb1[i]~dnorm(matmb1[materialb1[i]],sigmb1[materialb1[i]])}

coeffb1 <-(LSVEC-NBS19)/(matmb1[1]-matmb1[2])

additb1<-LSVEC-coeffb1*matmb1[1]

matnormb1<-matmb1[3]*coeffb1+additb1

difb1<-matmb1[1]-matnormb1

for(i in 1:3){matmg1[i]~dnorm(0,1.0E-5)

sigmg1[i]~dgamma(1.0E-5,1.0E-5)

}

for( i in 1:29){measg1[i]~dnorm(matmg1[materialg1[i]],sigmg1[materialg1[i]])}

coeffg1 <-(LSVEC-NBS19)/(matmg1[1]-matmg1[2])

additg1<-LSVEC-coeffg1*matmg1[1]

matnormg1<-matmg1[3]*coeffg1+additg1

difg1<-matmg1[1]-matnormg1

}

list(sigmb1=c(1,1,1),sigmg1=c(1,1,1))

**Appendix C: OpenBUGS codes to obtain the 18 individual NBS22 values in Table 8**

{NBS22~dnorm(-30.03,400)

LSVEC~dnorm(-46.6,434)

USGS44~dnorm(-42.21,434)

NBS19~dnorm(1.95,1111)

IAEA~dnorm(2.46,10000)

dif<-LSVEC-USGS44

for( i in 1:10){

for(j in 1:3){matmb2[j,i]~dnorm(0,1.0E-5)

sigmb2[j,i]~dgamma(1.0E-5,1.0E-5)

}}

for( i in 1:300){measb2[i]~dnorm(matmb2[materialb2[i],dayb2[i]],sigmb2[materialb2[i],dayb2[i]])}

for(i in 1:8){coeffb2[i]<-(NBS22-IAEA)/(matmb2[3,i]-matmb2[2,i])

additb2[i]<-NBS22-coeffb2[i]*matmb2[3,i]

}

for(i in 9:10){coeffb2[i]<-(NBS22-NBS19)/(matmb2[3,i]-matmb2[2,i])

additb2[i]<-NBS22-coeffb2[i]*matmb2[3,i]

}

for(i in 1:10){matnormb2[i]<-matmb2[1,i]*coeffb2[i]+additb2[i]

LSVECt8b2[i]<-matnormb2[i]+dif

scaleb2[i]<-(-46.6-1.95)/(LSVECt8b2[i]-NBS19)

addb2[i]<-NBS19- NBS19*scaleb2[i]

NBS22adjb2[i]<-NBS22*scaleb2[i]+addb2[i]

}

for( i in 1:8){

for(j in 1:3){matmr2[j,i]~dnorm(0,1.0E-5)

sigmr2[j,i]~dgamma(1.0E-5,1.0E-5)

}}

for( i in 1:153){measr2[i]~dnorm(matmr2[materialr2[i],dayr2[i]],sigmr2[materialr2[i],dayr2[i]])}

for(i in 1:8){coeffr2[i]<-(NBS22-NBS19)/(matmr2[3,i]-matmr2[2,i])

additr2[i]<-NBS22-coeffr2[i]*matmr2[3,i]

}

for(i in 1:8){matnormr2[i]<-matmr2[1,i]*coeffr2[i]+additr2[i]

LSVECt8r2[i]<-matnormr2[i]+dif

scaler2[i]<-(-46.6-1.95)/(LSVECt8r2[i]-NBS19)

addr2[i]<-NBS19- NBS19*scaler2[i]

NBS22adjr2[i]<-NBS22*scaler2[i]+addr2[i]}

}

list(

sigmb2=structure(.Data=c(1,1,1,1,1,1,1,1,1,1,1,1,1,1,1,1,1,1,1,1,1,1,1,1,1,1,1,1,1,1),.Dim=c(3,10)),

sigmr2=structure(.Data=c(1,1,1,1,1,1,1,1,1,1,1,1,1,1,1,1,1,1,1,1,1,1,1,1),.Dim=c(3,8)))

**Appendix D: Multivariate Gaussian meta-analysis model programed in OpenBUGS to obtain**

**the consensus USGS44 value for Table 8**

rrf[1,1:18]~dmnorm(rfm[],tau[,])

for(i in 1:18){rfm[i]~dnorm(murf,rfsig)}

murf~dnorm(0,1.0E-5)

xinsw~dnorm(0,0.0016)I(0.001,)

chsqnsw~dgamma(0.5,0.5)

rfsig<-xinsw/sqrt(chsqnsw)

}

Initial values:

list(rfsig=1)

Data:

list(tau=structure(.Data=c( 537.677,-21.6878,-26.9347,-14.7539,-32.1554,-50.293,-35.1325,-30.517,-22.9947,-64.1791,

-25.5216,-38.022,-56.2775,-5.95485,-1.39222,-10.4137,-13.4469,-80.9951,-21.6878,621.294,-17.948,-42.8813,-43.282,

-51.2919,-49.1535,-65.2278,-24.9943,-72.4756,-23.5174,-38.1874,-51.4748,-8.33417,-9.13575,-12.4432,-21.6877,

-78.7561,-26.9347,-17.948,779.767,-49.2982,-58.0149,-52.3416,-84.6458,-98.5569,-41.7779,-66.2443,-44.7427,

-39.3847,-65.5107,-4.05617,-9.79572,-24.9566,-27.7838,-106.599,-14.7539,-42.8813,-49.2982,1278.94,-87.2368,

-106.145,-118.862,-142.441,-49.5714,-128.998,-49.0682,-78.3068,-99.4167,-2.91468,-3.82665,-10.865,-46.3591,

-170.618,-32.1554,-43.282,-58.0149,-87.2368,948.451,-83.759,-89.378,-106.538,-48.8418,-89.5136,-18.2682,-70.171,

-95.7738,-2.21614,-4.57809,-26.2823,-33.0089,-135.704,-50.293,-51.2919,-52.3416,-106.145,-83.759,1563.94,-129.702,

-168.148,-72.628,-175.81,-58.5259,-113.569,-122.23,-4.57664,-14.441,-42.4389,-35.916,-216.056,-35.1325,-49.1535,

-84.6458,-118.862,-89.378,-129.702,1629.57,-182.611,-95.8527,-177.246,-59.578,-104.884,-149.57,-6.53948,-15.1573,

-46.6808,-21.7804,-195.803,-30.517,-65.2278,-98.5569,-142.441,-106.538,-168.148,-182.611,1877.52,-83.9765,

-179.578,-75.5446,-118.205,-208.14,-8.85774,-14.7121,-51.2713,-57.1217,-228.444,-22.9947,-24.9943,-41.7779,

-49.5714,-48.8418,-72.628,-95.8527,-83.9765,956.417,-115.196,-50.1273,-85.1364,-109.701,-5.69533,-12.3201,

-38.2996,-23.13,-152.364,-64.1791,-72.4756,-66.2443,-128.998,-89.5136,-175.81,-177.246,-179.578,-115.196,1996.53,

-70.5826,-143.667,-235.391,-9.93937,-19.7706,-51.9313,-74.9609,-268.957,-25.5216,-23.5174,-44.7427,-49.0682,

-18.2682,-58.5259,-59.578,-75.5446,-50.1273,-70.5826,804.97,-67.581,-85.0278,-2.0892,-8.53435,-31.4974,-31.362,

-150.022,-38.022,-38.1874,-39.3847,-78.3068,-70.171,-113.569,-104.884,-118.205,-85.1364,-143.667,-67.581,1408.12,

-158.429,-7.07088,-5.51878,-46.6924,-39.376,-179.985,-56.2775,-51.4748,-65.5107,-99.4167,-95.7738,-122.23,-149.57,

-208.14,-109.701,-235.391,-85.0278,-158.429,1968.21,-16.3229,-24.9314,-51.8822,-74.7497,-307.229,-5.95485,

-8.33417,-4.05617,-2.91468,-2.21614,-4.57664,-6.53948,-8.85774,-5.69533,-9.93937,-2.0892,-7.07088,-16.3229,99.355,

-0.281308,-0.321913,-0.729855,-10.1151,-1.39222,-9.13575,-9.79572,-3.82665,-4.57809,-14.441,-15.1573,-14.7121,

-12.3201,-19.7706,-8.53435,-5.51878,-24.9314,-0.281308,191.714,-7.55486,-3.3007,-38.8521,-10.4137,-12.4432,

-24.9566,-10.865,-26.2823,-42.4389,-46.6808,-51.2713,-38.2996,-51.9313,-31.4974,-46.6924,-51.8822,-0.321913,

-7.55486,552.966,-22.1771,-72.2666,-13.4469,-21.6877,-27.7838,-46.3591,-33.0089,-35.916,-21.7804,-57.1217,-23.13,

-74.9609,-31.362,-39.376,-74.7497,-0.729855,-3.3007,-22.1771,611.388,-92.8277,-80.9951,-78.7561,-106.599,-170.618,

-135.704,-216.056,-195.803,-228.444,-152.364,-268.957,-150.022,-179.985,-307.229,-10.1151,-38.8521,-72.2666,

-92.8277,2528.28),.Dim=c(18,18)),rrf=structure(.Data=c(-42.259,-42.327,-42.317,-42.275,-42.317,-42.324,-42.186,-42.209,-42.365,-42.309,-42.247,-42.278,-42.267,-42.249,-42.24,-42.258,-42.238,-42.254),.Dim=c(1,18)))
